# Integrated metabolomics and computational analysis suggest that a *Sanghuangporus vaninii*-based formulation alleviates T2DM in mice and modulates hepatic morphine-3-glucuronide axis

**DOI:** 10.3389/fnut.2026.1826245

**Published:** 2026-05-08

**Authors:** Zifeng Huang, Ying Chen, Yanfa Lin, Yueguang Wang, Tiantian Li, Xiaodong Ge, Bin Liu, Zirui Huang

**Affiliations:** 1 College of Food Science, Fujian Agriculture and Forestry University, Fuzhou, China; 2 Department of Traditional Chinese Medicine, Fujian Agriculture and Forestry University Hospital, Fuzhou, China; 3 National Engineering Research Center of JUNCAO Technology, Fujian Agriculture and Forestry University, Fuzhou, China; 4 Fuzhou Institute of Oceanography, Minjiang University, Fuzhou, China; 5 College of Marine and Bioengineering, Yancheng Institute of Technology, Yancheng, China

**Keywords:** endogenous morphine-3-glucuronide, insulin resistance, liver metabolomics, network pharmacology, *Sanghuangporus vaninii*

## Abstract

**Introduction:**

This study aimed to investigate the therapeutic material basis and mechanisms of a *Sanghuangporus vaninii*-based functional food formulation (FSV) in type 2 diabetes mellitus (T2DM) using an integrated approach combining chemical profiling, metabolomics, and computational biology.

**Methods:**

Chemical profiling of FSV was performed using UPLC-QTOF/MS, followed by network pharmacology to identify core bioactive constituents. The therapeutic effects of FSV were evaluated in a T2DM mouse model (*n* = 10 per group, 4-week intervention, with metformin as a positive control) by assessing hyperglycemia, dyslipidemia, and hepatic steatosis. Hepatic metabolomics was conducted to explore metabolic changes, and molecular docking was employed to investigate potential interactions between key metabolites and diabetes-related targets.

**Results:**

UPLC-QTOF/MS analysis identified 766 compounds, with 22 core flavonoids (e.g., apigenin, tangeritin) selected via network pharmacology. In T2DM mice, FSV intervention significantly ameliorated hyperglycemia, dyslipidemia, and hepatic steatosis. Liver metabolomics revealed targeted modulation of the glucagon signaling pathway, marked by reduced levels of β-D-fructose 6-phosphate and oxaloacetate. Notably, endogenous morphine-3-glucuronide (M3G) was identified as a notable hepatic metabolite. Molecular docking suggested that M3G may potentially interact with PIK3CA/EGFR based on computational prediction, while FSV was associated with reduced hepatic M3G levels in T2DM mice. However, these *in silico* findings require experimental validation.

**Conclusion:**

These findings suggest that FSV not only delivers bioactive flavonoids but also modulates specific endogenous metabolites such as M3G, offering new chemical and metabolic insights into functional food-based diabetes therapy.

## Graphical abstract

**Figure:**
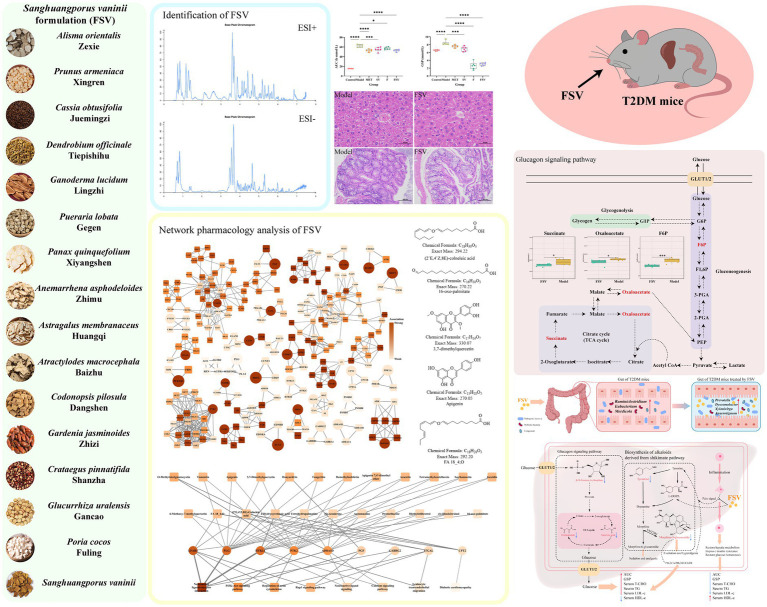

## 1 Introduction

Diabetes mellitus, a chronic metabolic disorder defined by persistent hyperglycemia, has emerged as a major global public health challenge with significant social and economic implications. According to the International Diabetes Federation, the global prevalence of diabetes among individuals aged 20–79 reached 589 million in 2024, with projections indicating it will surpass 850 million by 2050 (1). Type 2 diabetes mellitus (T2DM), the most prevalent form, is primarily driven by insulin resistance and partial pancreatic β-cell dysfunction. T2DM complications, including diabetic nephropathy, retinopathy, neuropathy, and cardiovascular diseases, severely affect patients’ quality of life and impose a substantial economic burden on healthcare systems worldwide (2).

Current pharmacological treatments for T2DM predominantly include metformin, sulfonylureas, SGLT2 inhibitors, and GLP-1 receptor agonists (3). These medications rapidly lower blood glucose through single-target mechanisms but are associated with side effects such as diarrhea, gastrointestinal discomfort, and acute hypoglycemia. Prolonged use may also lead to drug resistance or secondary failure, limiting their long-term efficacy (4). Consequently, there is an urgent need for low-toxicity, low-risk adjunctive therapies, with dietary interventions based on functional foods emerging as a promising research avenue. Recent evidence suggests that diabetes-induced metabolic stress may disrupt the endogenous opioid system in peripheral tissues, leading to the accumulation of atypical metabolites that impair normal signaling.

Functional foods, which contain bioactive components such as polysaccharides, flavonoids, and saponins, provide health benefits beyond basic nutrition (5). In recent years, these functional foods have garnered significant attention for their potential in managing chronic diseases. Unlike conventional pharmaceuticals, functional foods regulate physiological processes through multi-target mechanisms, making them ideal candidates for long-term adjunctive therapies for chronic conditions like T2DM (6, 7). Numerous studies have identified bioactive components in functional foods from medicinal fungi including *Alisma orientale* (Zexie), *Anemarrhena asphodeloides* (Zhimu), *Astragalus membranaceus* (Huangqi), *Atractylodes macrocephala* (Baizhu), *Cassia obtusifolia* (Juemingzi), *Codonopsis pilosula* (Dangshen), *Crataegus pinnatifida* (Shanzha), *Dendrobium officinale* (Tiepishihu), *Ganoderma lucidum* (Lingzhi), *Gardenia jasminoides* (Zhizi), *Glycyrrhiza uralensis* (Gancao), *Panax quinquefolium* (Xiyangshen), *Prunus armeniaca* (Xingren), *Poria cocos* (Fuling), and *Pueraria lobata* (Gegen), which exhibit biological activities beneficial for chronic disease management. For example, Huangqi polysaccharides and Tiepishihu polysaccharides enhance insulin sensitivity (8, 9); flavonoids from Shanzha and Gegen reduce serum triglycerides and cholesterol levels (10, 11); Zexie and Zhimu alleviate hepatic lipid accumulation (12, 13); triterpenoids from Lingzhi modulate immune function to mitigate chronic inflammation (14); glycyrrhizic acid from Gancao exhibits anti-inflammatory and antioxidant effects (15); saponins from Xiyangshen improve cardiomyocyte viability (16); and Xingren and Fuling enhance gut microbiota composition (17, 18).

Among functional foods, *Sanghuangporus vaninii*, an edible and medicinal fungus, has emerged as a promising dual-function candidate (19). Its bioactive substances-polysaccharides, phenolic compounds, and triterpenoids-contribute to its health benefits. *S. vaninii* shows considerable promise in T2DM management: its polysaccharides promote β-cell proliferation and insulin secretion, while its triterpenoids and phenolic compounds alleviate oxidative stress (20–22). These findings position *S. vaninii* as a valuable adjunctive functional food for T2DM. Therefore, combining *S. vaninii* with other functional foods may generate synergistic effects, enhancing glucose-lowering outcomes.

This study developed an *S. vaninii*-based functional food formulation (FSV) by incorporating *S. vaninii* extract (SV) into a basic functional food formulation (F) to explore its glucose-lowering mechanisms in T2DM mice. Using UPLC-QTOF/MS, network pharmacology, biochemical assays, liver metabolomics, and 16S rRNA sequencing of cecal content, we confirmed the efficacy of FSV and its correlations with gut microbiota and metabolism. The results suggest FSV as a promising T2DM-targeted functional food and provide preliminary information on its potential material basis within target tissues.

## 2 Materials and methods

### 2.1 Materials and reagents

The following medicinal herbs were supplied by Sichuan Guoqiangzhongyao Co., Ltd. (Chengdu, China): *Alisma orientale* (Zexie), *Anemarrhena asphodeloides* (Zhimu), *Astragalus membranaceus* (Huangqi), *Atractylodes macrocephala* (Baizhu), *Cassia obtusifolia* (Juemingzi), *Codonopsis pilosula* (Dangshen), *Crataegus pinnatifida* (Shanzha), *Dendrobium officinale* (Tiepishihu), *Ganoderma lucidum* (Lingzhi), *Gardenia jasminoides* (Zhizi), *Glycyrrhiza uralensis* (Gancao), *Panax quinquefolium* (Xiyangshen), *Prunus armeniaca* (Xingren), *Poria cocos* (Fuling), and *Pueraria lobata* (Gegen). *S. vaninii* was provided by the Hangzhou Academy of Agricultural Sciences (Hangzhou, China). All reagents used in this study were of analytical grade.

### 2.2 Preparation of F, SV, and FSV

The preparation process for the 16 functional foods was based on the solubility of their primary active ingredients as illustrated in Supplementary Figure S1. Initially, the 16 functional foods were ground into particles approximately the size of rice grains. Subsequently, Zexie, Xingren, Juemingzi, Tiepishihu, Lingzhi, and Gegen were then extracted with 70% ethanol at a ratio of 1:30 (m/v) via ultrasonic-assisted extraction at 60 °C for 1 h (300 W, 45 kHz), with extraction repeated twice (ethanol extraction). The residual material was subsequently extracted with ultrapure water at a ratio of 1:50 (m/v) at 60 °C for 1 h, followed by an increase in temperature to 100 °C for an additional 2 h. This process was repeated twice (water extraction). The four resulting extracts were combined and freeze-dried to obtain extract A (A). Meanwhile, Xiyangshen, Zhimu, Huangqi, Baizhu, Dangshen, Zhizi, Shanzha, Gancao, and Fuling were extracted with water to obtain extract B (B). *S. vaninii* underwent both ethanol and water extraction to obtain extract C (C), referred to as SV. Extracts A and B were then combined according to the recommended daily dosage for humans to prepare formulation F. Similarly, extracts A, B, and C were combined based on the recommended daily dosage to prepare the *S. vaninii*-based functional food formulation (FSV).

### 2.3 Principal nutrient content and UPLC-QTOF/MS analysis of FSV

Total phenolic content in FSV was determined using gallic acid (GAE) as the standard. The sample was mixed with Folin–Ciocalteu reagent and 7.5% Na_2_CO_3_ to facilitate the reaction. Total sugar content was assessed using glucose (Glu) as the standard, with the sample undergoing complete hydrolysis with concentrated sulfuric acid, followed by reaction with 6% phenol. Total flavonoid content was measured using rutin as the standard, where the sample was combined with 5% NaNO_2_ and then mixed with 10% Al(NO_3_)_3_. The reaction was continued with the addition of 4% NaOH and 70% ethanol. Finally, protein content in FSV was quantified using a BCA protein assay kit (Beyotime, China).

The composition of FSV was identified following the method described in a previous study (23), with compounds retained within a mass tolerance of 5 ppm (24). The raw data were converted to mzXML format using the MSConvert tool from the ProteoWizard software package (version 3.0.8789) and processed with R XCMS (version 3.12.0) for feature detection, retention time correction, and alignment. Data were normalized using the area normalization method to correct for systematic errors. Metabolite identification was performed based on accurate mass and MS/MS data, which were matched against several databases, including HMDB (25), MassBank^1^, LipidMaps^2^, mzCloud^3^, and KEGG (26). MS/MS data were compared with fragment ions and additional information in the database to enable comprehensive metabolite identification.

### 2.4 FSV network pharmacology analysis of FSV

The SMILES strings of all compounds were first obtained from PubChem (27), followed by evaluation of their biological activity using SwissADME (28). This process involved assessing their gastrointestinal absorption (GI) index and confirming that they passed at least three drug-likeness predictions. Potential targets for these active compounds were identified using SwissTargetPrediction (probability ≥ 0.1) (29). Known targets for T2DM were then gathered from major disease-related databases, including GeneCards (30), OMIM^4^, and DrugBank (31). Disease-related targets from GeneCards were filtered based on the median value, and all targets were combined, duplicates removed, and common targets identified by intersecting them with the potential targets of active compounds using Venny (version 2.1.0) (32). Protein–protein interaction (PPI) network analysis was performed on the common targets, setting a minimum required interaction score of 0.900. The MCODE tool in Cytoscape software was employed to analyze the PPI network and identify key targets (33). Gene Ontology (GO) functional enrichment and pathway enrichment analyses were then conducted on the key targets (34), with enrichment targets filtered by the median value. A network diagram was visualized to illustrate the relationships between active compounds, genes, and signaling pathways.

### 2.5 Animal experiments and biochemical index detection

All experimental animals were male, aged 6 weeks, SPF-grade ICR mice with an average weight of 26 ± 2 g, obtained from the Wu’s Experimental Animal Center (Fuzhou, China). Two types of diets were employed: a normal feed diet (NFD) containing 22.47% protein, 12.11% fat, and 65.42% carbohydrates, and a high-fat, high-sugar diet (HFD), comprising 15% sucrose, 15% lard, 1% cholesterol, 10% egg yolk powder, 0.2% bile salts, and 58.8% NFD. All mice were housed under controlled conditions (temperature 23 ± 2 °C, humidity 55 ± 5%, 12-h light/dark cycle), with ad libitum access to drinking water. After a one-week acclimatization period, during which all mice received the NFD, the mice were randomly assigned to either the Control group (Control, *n* = 10) or the modeling group (*n* = 50). Mice in the modeling group received a single intraperitoneal injection of streptozotocin (STZ) at a dose of 125 mg/kg, followed by a one-week HFD feeding to facilitate the establishment of T2DM (35). The Control group remained on the NFD throughout the entire study. Seven days after STZ injection, the fasting blood glucose (FBG) of the mice was measured, and the T2DM model was considered successfully established when FBG reached or exceeded 11.1 mmol/L. The 50 successfully modeled mice were individually numbered, and randomly assigned to five groups (*n* = 10 per group) using the Excel random number generator: Model, MET, SV, F, and FSV groups. During the subsequent 4-week intervention period, the Control group continued to receive the NFD, whereas the maintenance diet of 50 T2DM mice was completely replaced with NFD, because patients tend to control their diet once they have diabetes. During the intervention period, each group of mice received oral gavage at consistent times and locations on a daily basis. Mice in Control and Model received 200 μL of physiological saline by oral gavage, while those in the MET group were administered 200 μL of 200 mg/kg metformin. The SV, F, and FSV groups received 200 μL of 0.4 g/kg *S. vaninii* extract, 7.7 g/kg F, and 8.1 g/kg FSV, respectively, by oral gavage. The dosing calculations were based on the recommended daily intake, using the formula: medicinal mushroom weight × extraction yield, with FSV composed of SV and F in a 1:1 ratio. On the 42nd day, an oral glucose tolerance test (OGTT) was conducted on all mice, and the following day, all mice were anesthetized with an intraperitoneal injection of 1% pentobarbital sodium, and blood samples were collected via exsanguination. Serum, liver, pancreas, cecum, and cecal contents were collected for further analysis. Biochemical indices, including glycosylated serum protein (GSP), total cholesterol (T-CHO), total triglycerides (TG), and high- and low-density lipoprotein cholesterol (HDL-c, LDL-c), were assessed using biochemical kits from Nanjing Jiancheng Bioengineering Institute Co., Ltd. (Nanjing, China). For the biochemical indices, a double-blind analysis was performed. Specifically, a third person who was unaware of the group allocation randomly selected six mice from each group using a random number table. No mice were excluded or died prior to selection. The entire study was in strict accordance with the ARRIVE guidelines 2.0. The study adhered to the laboratory animal welfare standards and ethical review procedures of the College of Food Science, Fujian Agriculture and Forestry University (No. FS-2021-0430).

### 2.6 Histopathological observation of organs

Similarly, a double-blind histological examination was performed. Fresh liver and cecal tissues were fixed in 4% paraformaldehyde solution and subsequently embedded in paraffin. Sections of 4 μm thickness were cut, stained with H&E, and examined for morphological alterations under a microscope (Optical microscope, SMZ18).

### 2.7 Metabolomic analysis of liver differential metabolites

Similarly, a double-blind analysis of the liver metabolome was conducted. LC analysis was conducted using a Vanquish UHPLC System (Thermo Fisher Scientific, United States) equipped with an ACQUITY UPLC® HSS T3 column (2.1 × 100 mm, 1.8 μm, Waters, Milford, MA, United States). Quality control (QC) samples were prepared by pooling equal aliquots of all study samples and were injected at regular intervals (every 10 injections) throughout the analytical run to monitor instrument stability and signal drift. Samples were injected in a randomized order to minimize batch effects. The column temperature was maintained at 40 °C, with a flow rate of 0.3 mL/min and an injection volume of 2 μL. For LC-ESI (+)-MS, the mobile phases consisted of 0.1% formic acid in acetonitrile (v/v, B2) and 0.1% formic acid in water (v/v, A2). For LC-ESI (−)-MS, the mobile phases included acetonitrile (B3) and 5 mM ammonium formate (A3). Both methods employed the same gradient elution: 10% B2/B3 for 0–1 min, increasing to 98% over 1–5 min, maintaining at 98% for 5–6.5 min, decreasing back to 10% in 0.1 min (6.5–6.6 min), and holding at 10% until 8 min for re-equilibration (36). Mass detection was carried out using a Thermo Fisher Orbitrap Exploris 120 mass spectrometer with an ESI source in Full MS-ddMS2 mode. The MS parameters were set as follows: sheath gas pressure 40 arb, auxiliary gas flow 10 arb, capillary temperature 325 °C, and spray voltage of 3.50 kV for ESI(+) and −2.50 kV for ESI(−). The MS1 scan range was m/z 100–1,000 with a 60,000 FWHM resolving power, and four data-dependent scans per cycle. For MS/MS, the resolution was set at 15,000 FWHM, with a normalized collision energy of 30% and automatic dynamic exclusion time (10 ppm) (37). Instrument stability was further confirmed by monitoring the retention time and peak area variations of QC samples, which showed a relative standard deviation (RSD) of <15% for the majority of features. Liver metabolites were identified following the procedure outlined in Section 2.2. Principal component analysis (PCA), orthogonal partial least squares discriminant analysis (OPLS-DA), and permutation tests were performed using SIMCA software (version 14.1) to reduce the dimensionality of the data. The model’s performance was assessed for overfitting via permutation testing with 200 permutations. OPLS-DA was used to calculate the variable importance in projection (VIP), while fold change analysis was employed to assess group differences. For differential metabolite screening, metabolites were considered statistically significant if the *p*-value was less than 0.05 and the VIP value exceeded 1. To account for multiple comparisons, the false discovery rate (FDR) was controlled using the Benjamini–Hochberg method, and only metabolites with an FDR-adjusted *p*-value < 0.05 were retained. KEGG pathway enrichment analysis was performed based on the hypergeometric distribution test, and topological analysis was conducted using betweenness centrality. Pathway *p*-values were adjusted for multiple comparisons using the FDR method, and pathways with an adjusted *p*-value < 0.05 were considered significantly enriched.

### 2.8 High-throughput sequencing analysis

Total DNA was extracted from 0.2 g of frozen cecal contents using the Magbeads FastDNA Kit for Soil (116564384) (MP Biomedicals, CA, United States). The V3–V4 region was amplified with the following primers: forward primer 338F (5’-ACTCCTACGGGAGGCAGCA-3′) and reverse primer 806R (5’-GGACTACHVGGGGTWTCTAAT-3′). The resulting 16S rRNA sequences were analyzed. Library construction was performed using the TruSeq Nano DNA LT Library Prep Kit, and library quantification was carried out on the Qubit 4, and PCR-enriched fragments were quality-checked using the Agilent 2,100. The pooled library (10 nM) was diluted to the appropriate concentration and sequenced in 2 × 250 bp paired-ends on an Illumina NovaSeq platform with the NovaSeq 6,000 SP Reagent Kit. Processed sequence data were compared to the Greengenes 13.8 database for microbial identification at each taxonomic level across the groups. Venn diagrams were generated to visualize shared and unique microorganisms between samples or populations, independent of their relative abundance. This analysis was conducted using R software (version 4.3.3).

### 2.9 Statistical analysis

Data are presented as mean ± SD. The box plot was created using GraphPad Prism (version 8.0), and the heatmap was generated with TBtools II (38). The association network was visualized using Cytoscape (version 3.7.1). For biochemical indices, one-way ANOVA followed by Tukey’s *post hoc* test was used to compare the mean values of the Model group against each of the other groups. For gut microbiota analysis, the non-parametric Kruskal–Wallis rank sum test was performed, and taxa with a LDA score greater than 2.5 were considered as significantly influential species. Statistical significance was determined as follows: * for *p* < 0.05, ** for *p* < 0.01, *** for *p* < 0.001, **** for *p* < 0.0001. *Post hoc* power analysis showed power > 0.40 for all primary endpoints (Supplementary Table S1).

## 3 Results

### 3.1 The main compounds of FSV and its network pharmacology analysis

The nutritional composition of FSV is summarized in Table 1. The formulation contains total phenolic content of 0.0814 ± 0.0013 mg GAE/mg, total sugars at 0.7112 ± 0.0125 mg Glu/mg, total flavonoids quantified at 0.0163 ± 0.0020 mg Rutin/mg, and protein levels of 0.0186 ± 0.0017 mg BSA/mg. These results provide a comprehensive overview of the primary nutritional components in FSV.

**Table 1:** Principal nutrient content in FSV.

Name	Total phenolic content mg GAE/mg	Total sugar content mg Glu/mg	Total flavonoid content mg Rutin/mg	Protein content mg BSA/mg
FSV	0.0814 ± 0.0013	0.7112 ± 0.0125	0.0163 ± 0.0020	0.0186 ± 0.0017

**Figure 1:**
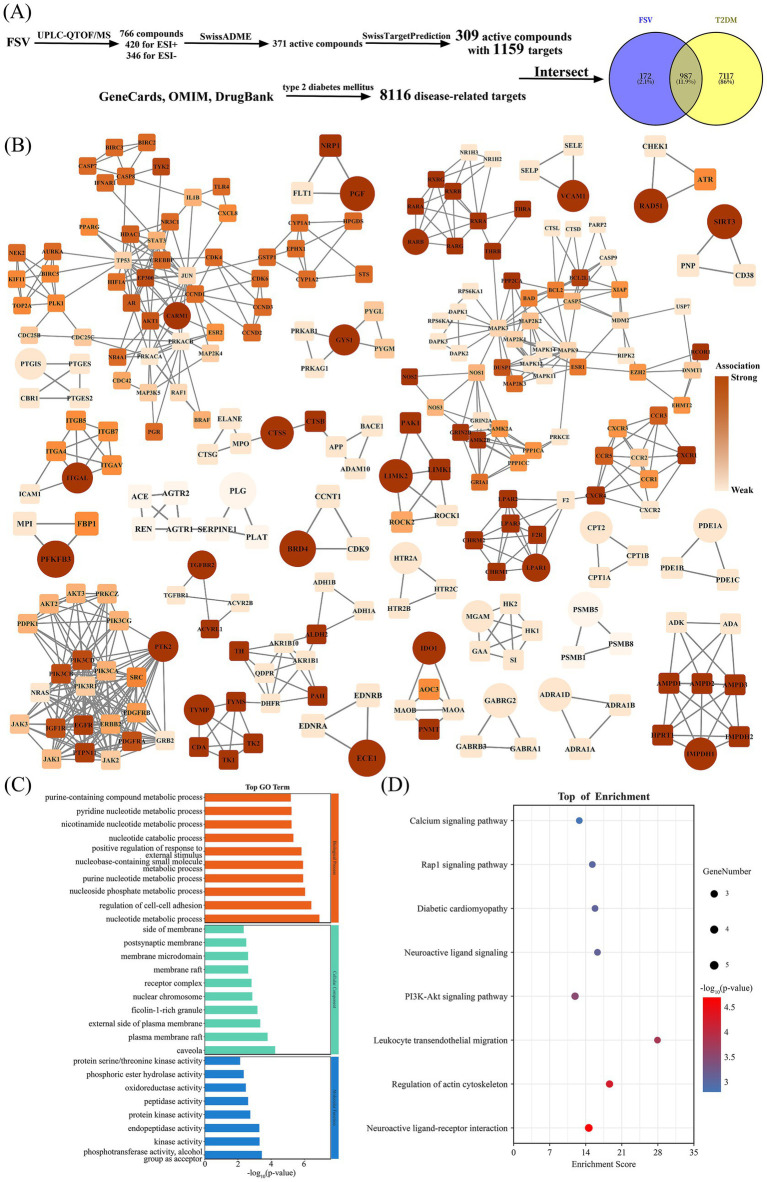
Comprehensive multi-omics analysis of FSV: component identification, target prediction, and protein interaction network profiling. **(A)** Identification of components in FSV, prediction of their targets of active compounds, and intersection analysis with T2DM-related targets. **(B)** Protein–protein interaction (PPI) network analysis of intersected targets. The depth of color indicates the strength of the association. Ellipse represents the key targets in the target relationships of each group. **(C)** GO functional enrichment analysis of FSV. **(D)** KEGG pathway enrichment analysis of FSV.

As shown in Figure 1A, the UPLC-QTOF/MS analysis identified 766 compounds in FSV. The ion base peak profile of FSV is shown in Supplementary Figure S2. SwissADME predictions identified 371 bioactive compounds, and SwissTargetPrediction narrowed this down to 309 compounds associated with valid biological targets, resulting in a cumulative total of 1,159 potential targets. A search for “type 2 diabetes mellitus” retrieved 8,116 disease-related targets from three databases. By intersecting these disease targets with the potential targets of FSV, 987 common targets were identified. PPI network analysis of these common targets revealed 28 gene subnetworks, with key targets illustrated in Figure 1B. Notable key targets include the alpha-1D adrenergic receptor (ADRA1D), bromodomain-containing protein 4 (BRD4), and coactivator-associated arginine methyltransferase 1 (CARM1). GO enrichment analysis of these 28 key targets identified significant associations with 118 biological processes (BP), 13 cellular components (CC), 8 molecular functions (MF), and 9 KEGG signaling pathways. The key biological pathways and processes are shown in Figures 1C,D. The BP analysis indicates enrichment in processes such as nucleotide metabolism, regulation of cell–cell adhesion, and nucleoside phosphate metabolism. The CC analysis suggests these targets are predominantly located in cellular structures like caveolae, plasma membrane rafts, and the external side of the plasma membrane. The MF analysis highlights activities such as phosphotransferase activity, alcohol group acceptance, kinase activity, and endopeptidase activity. KEGG pathway analysis reveals significant enrichment in pathways like neuroactive ligand-receptor interactions, regulation of the actin cytoskeleton, and leukocyte transendothelial migration.

**Figure 2:**
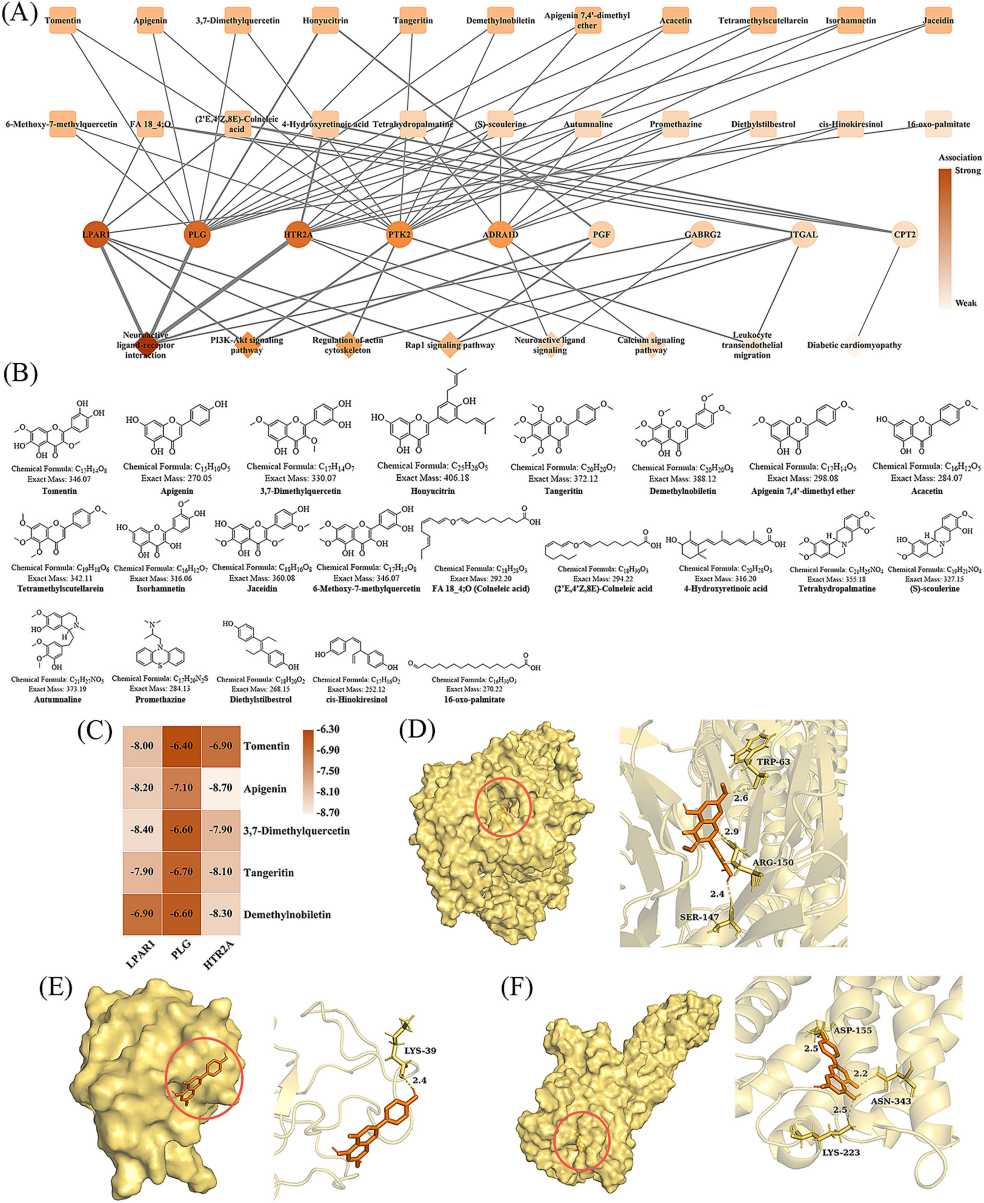
Network analysis and molecular docking. **(A)** Network diagram of active compounds, genes, and signaling pathways (the depth of color and the thickness of the line indicate the strength of the association round rectangle stands for active compounds, ellipse for genes, and diamond for signaling pathways). **(B)** Chemical structures of the active compounds in **(A)**. **(C)** Minimum binding energy of molecular docking (kcal/mol). **(D)** Pair docking visualization of LPAR1 and 3,7-dimethylquercetin. **(E)** Pair docking visualization of PLG and apigenin. **(F)** Pair docking visualization of HTR2A and apigenin. Notes: The site of molecular docking is marked with a red circle in **(D–F)**.

Subsequently, nine core targets—lysophosphatidic acid receptor 1 (LPAR1), plasminogen (PLG), 5-hydroxytryptamine receptor 2A (HTR2A), focal adhesion kinase 2 (PTK2), ADRA1D, placental growth factor (PGF), gamma-aminobutyric acid receptor subunit gamma-2 (GABRG2), integrin alpha-L (ITGAL), and carnitine palmitoyltransferase 2 (CPT2)—were identified by intersecting the 28 key targets with those involved in the enriched signaling pathways. A network diagram was constructed to visualize the interactions among bioactive compounds, core genes, and signaling pathways, integrating 22 bioactive compounds and eight signaling pathways related to these core targets (Figure 2A). The 22 bioactive compounds in FSV have the potential to regulate T2DM through nine targets (e.g., LPAR1, PLG, and HTR2A) and eight pathways (e.g., neuroactive ligand-receptor interactions, PI3K-Akt signaling pathway, and actin cytoskeleton regulation). The chemical structures of these 22 bioactive compounds are depicted in Figure 2B, primarily consisting of flavonoids and fatty acids. The simple comparison chart of these 22 compounds can be found in Supplementary Figure S3.

The ADME characteristics of the 22 bioactive compounds are summarized in Table 2, with a detailed list provided in the Supplementary Table S2. In this study, “High GI absorption” refers to the favorable gastrointestinal absorption capabilities of the active compounds, while “BBB permeant” indicates their ability to cross the blood–brain barrier. Additionally, “P-gp substrate” denotes whether a compound is susceptible to efflux by P-glycoprotein. Drug-likeness is assessed using established criteria from Lipinski, Ghose, Veber, Egan, and Muegge, which provide a framework for evaluating the suitability of compounds. The number of rules satisfied by a compound indicates its overall drug-likeness. Table 2 demonstrates that most bioactive compounds meet the necessary criteria. Consequently, compounds exhibiting high gastrointestinal absorption, classified as non-P-gp substrate characteristics, and compliance with five drug-likeness rules were selected for subsequent molecular docking simulations. These compounds include Tomentin, Apigenin, 3,7-Dimethylquercetin, Tangeritin, and Demethylnobiletin. Further details regarding small molecule ligands, core targets, and pathways involved in the network pharmacology analysis of FSV and T2DM are provided in Table 3.

**Table 2:** ADME information of the 22 bioactive compounds in Figure 2A.

Name	GI absorption	BBB permeant	P-gp substrate	Lipinski	Ghose	Veber	Egan	Muegge
Tomentin	High	No	No	Yes	Yes	Yes	Yes	Yes
Apigenin	High	No	No	Yes	Yes	Yes	Yes	Yes
3,7-Dimethylquercetin	High	No	No	Yes	Yes	Yes	Yes	Yes
Honyucitrin	High	No	No	Yes	Yes	Yes	Yes	No
Tangeritin	High	Yes	No	Yes	Yes	Yes	Yes	Yes
Demethylnobiletin	High	No	No	Yes	Yes	Yes	Yes	Yes
Apigenin 7,4′-dimethyl ether	High	Yes	No	Yes	Yes	Yes	Yes	Yes
Acacetin	High	No	No	Yes	Yes	Yes	Yes	Yes
Tetramethylscutellarein	High	Yes	No	Yes	Yes	Yes	Yes	Yes
Isorhamnetin	High	No	No	Yes	Yes	Yes	Yes	Yes
Jaceidin	High	No	No	Yes	Yes	Yes	Yes	Yes
6-Methoxy-7-methylquercetin	High	No	No	Yes	Yes	Yes	Yes	Yes
FA 18_4; O	High	Yes	No	Yes	Yes	No	Yes	No
(2′E,4’Z,8E)-Colneleic acid	High	Yes	No	Yes	Yes	No	Yes	No
4-Hydroxyretinoic acid	High	Yes	No	Yes	Yes	Yes	Yes	Yes
Tetrahydropalmatine	High	Yes	Yes	Yes	Yes	Yes	Yes	Yes
(S)-scoulerine	High	Yes	Yes	Yes	Yes	Yes	Yes	Yes
Autumnaline	High	Yes	No	Yes	Yes	Yes	Yes	Yes
Promethazine	High	Yes	No	Yes	Yes	Yes	Yes	Yes
Diethylstilbestrol	High	Yes	No	Yes	Yes	Yes	Yes	No
*cis*-Hinokiresinol	High	Yes	No	Yes	Yes	Yes	Yes	Yes
16-oxo-palmitate	High	Yes	No	Yes	Yes	No	Yes	No

**Table 3:** Information of small molecule ligands, core targets, and pathways in network pharmacology analysis of FSV and T2DM.

Name	Betweenness	Closeness	Degree
Tomentin	0.00522804	0.29057592	2
Apigenin	0.00522804	0.29057592	2
3,7-Dimethylquercetin	0.00522804	0.29057592	2
Tangeritin	0.00522804	0.29057592	2
Demethylnobiletin	0.00522804	0.29057592	2
LPAR1	0.2056044	0.35126582	31
PLG	0.29292166	0.3404908	22
HTR2A	0.41824666	0.33841463	7
Neuroactive ligand-receptor interaction	0.3740083	0.375	5
PI3K-Akt signaling pathway	0.1202287	0.31534091	4

Figure 2C presents a heatmap illustrating the minimum binding energies of the five bioactive compounds binding to LPAR1, PLG, and HTR2A. All observed binding energies were less than −6 kcal/mol, indicating strong interactions between these compounds and their respective protein targets. Figures 2D–F depicts the docking positions of LPAR1 with 3,7-dimethylquercetin, the interaction between PLG and apigenin, and the docking details of HTR2A with apigenin. Notably, the compounds interact with the active pockets of their targets in an embedded manner, supporting the docking results. These findings suggest that FSV may exert its effects on T2DM through its flavonoid compounds by targeting LPAR1 and HTR2A. This interaction may hypothetically modulate neuroactive ligand-receptor interactions and influence key signaling pathways such as the PI3K-Akt pathway, which could be associated with the improvement of T2DM.

Network pharmacology analysis has revealed that the 22 core bioactive compounds in FSV regulate T2DM through nine key targets and eight primary signaling pathways, with flavonoids playing a particularly significant role. Molecular docking studies reveal that all bioactive compounds form stable interactions with their respective protein targets, as demonstrated by their minimum binding energies consistently falling below −6.0 kcal/mol. For example, apigenin has been shown to mitigate metabolic syndrome induced by a high-fat, high-fructose diet by reducing pro-inflammatory cytokines, enhancing insulin sensitivity, and lowering FBG levels (39). Additionally, 3,7-dimethylquercetin exhibits protective effects on liver tissue by modulating cell apoptosis (40). The neuroactive ligand-receptor interaction pathway, which is essential for neural function, involves LPAR1, a key receptor that regulates cell proliferation, migration, survival, and apoptosis. Inhibition of LPAR1 can lead to various neural disorders (41). Furthermore, HTR2A has been found to suppress neuroinflammation (42), highlighting the importance of neural signal transduction in the mechanism by which FSV modulates T2DM. The PI3K-Akt signaling pathway, central to insulin signal transduction and cellular energy regulation, is activated to reduce inflammation, improve liver morphology, and alleviate insulin resistance (43). PTK2, by upregulating this pathway, facilitates the repair of intestinal epithelial cell damage (44). In addition, inflammation associated with T2DM can result in the overactivation of PGF, which promotes angiogenesis and subsequently triggers the PI3K-Akt pathway, contributing to the reduction of vascular inflammation (45). The calcium signaling pathway, which is often dysregulated in diabetic patients, negatively affects cardiovascular health (46, 47). Chronic hyperglycemia leads to the upregulates ADRA1D, contributing to vascular damage (48). These findings suggest that the flavonoids in FSV may restore insulin signaling by modulating neural signal transduction, repairing damaged cells to alleviate vascular inflammation, and promoting the regeneration of the liver and cecum.

### 3.2 Effect of SV, F, and FSV on biochemical indexes, liver, and cecum of T2DM mice

**Figure 3:**
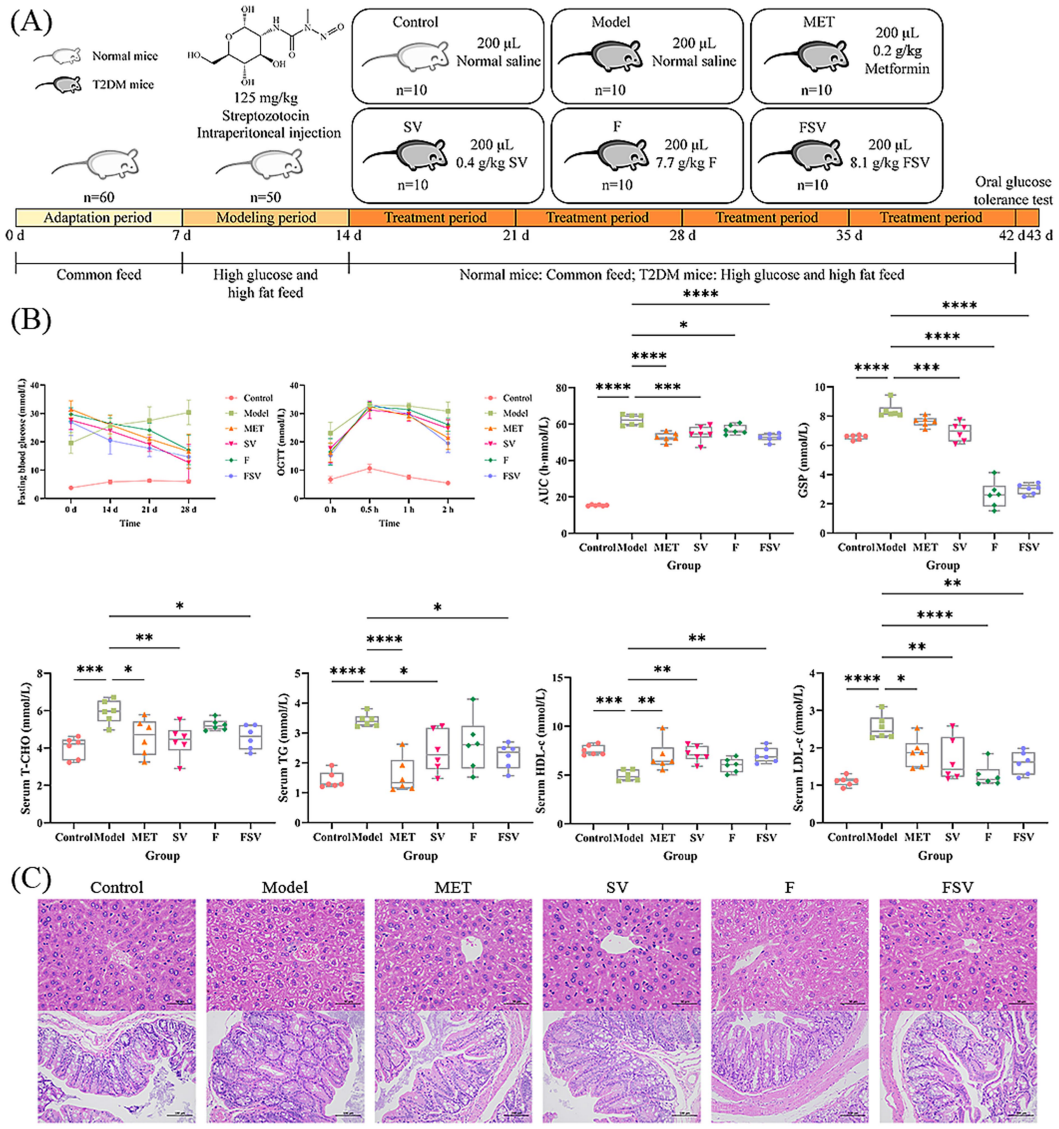
Effects of SV, F, and FSV intervention on the T2DM mice during the experimental period. **(A)** Schematic diagram of animal experiment. **(B)** Boxplots showing change in biochemical indexes of FBG, OGTT, AUC, GSP, serum T-CHO, serum TG, serum HDL-c, and serum LDL-c. **(C)** Histopathological analysis of liver (400×, 50 μm) and cecum (200×, 100 μm). Data are mean ± SD (*n* = 6). One-way ANOVA followed by Tukey’s *post hoc* test was used for comparisons among groups. *p < 0.05, **p < 0.01, ***p < 0.001, ****p < 0.0001 compared with the Model group.

The experimental procedures conducted in this study are outlined in Figure 3A. As shown in Figure 3B, significant biochemical differences were observed between T2DM mice in the Model group and normal mice (*p* < 0.001). Treatment with SV, F, and FSV resulted in a significant reduction in FBG levels in T2DM mice. Furthermore, FSV notably improved glucose tolerance and led to a substantial decrease in serum GSP and LDL-c levels (*p* < 0.01). Additionally, FSV significantly lowered serum T-CHO and TG levels (*p* < 0.01) while increasing serum HDL-c levels (*p* < 0.01). As shown in Figure 3C, distinct morphological differences were observed between the livers and ceca of T2DM mice and their normal counterparts. In T2DM mice, hepatocytes exhibited disorganized arrangements, accompanied by substantial cellular damage. Moreover, the cecum’s mucosal architecture was compromised, with villi showing shortening and distortion, indicative of an inflammatory response. Treatment with metformin, SV, F, and FSV resulted in significant improvements in liver and cecal morphology compared to the Model group. Specifically, the SV-treated group showed well-organized hepatocytes with reduced hepatic injury. Cecal damage was alleviated, and villus morphology showed signs of recovery. In the F group, hepatocyte damage was partially mitigated, though some disorganization remained. The cecal structure showed clearer architecture with partial recovery of villus morphology. In contrast, the FSV group exhibited well-aligned hepatocytes, reduced inflammation, and a more defined and ordered cecal mucosal structure, with villi resembling the morphology of normal tissue. These results demonstrate that FSV has hypoglycemic and lipid-lowering effects in T2DM mice and provide a basis for further investigation into whether these effects are associated with the predicted targets and pathways.

This study explored the therapeutic effects of SV, F, and FSV on T2DM. Based on serum biochemical markers, FSV, which includes SV, demonstrated a more favorable regulatory impact on glucose metabolism-related indicators compared to F. Intervention with FSV resulted in lower T-CHO and TG levels and higher HDL-c levels in T2DM mice, likely due to FSV’s enhanced ability to improve insulin resistance. The OGTT further indicates that FSV enhances glucose uptake and utilization efficiency in T2DM mice. Histopathological analysis reveals that FSV exerts superior reparative effects on hepatocyte and cecal morphology compared to F. Consequently, it is hypothesized that FSV regulates glucose metabolism more effectively and exhibits stronger anti-inflammatory effects on the inflamed liver and cecum, which is consistent with findings from the study by Dai et al. (49).

### 3.3 Effects of SV, F, and FSV on gut microbiota in T2DM mice

**Figure 4:**
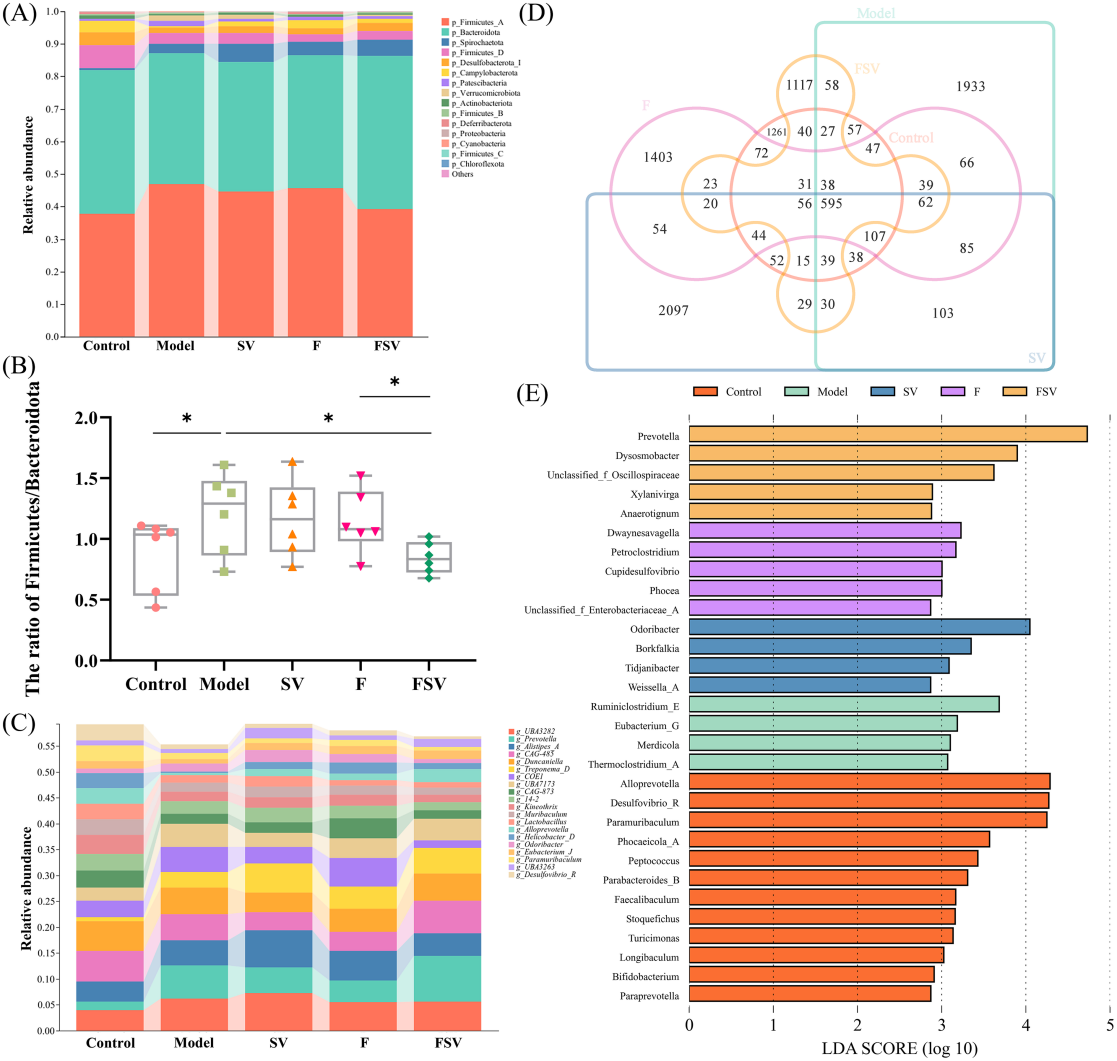
Effects of SV, F, and FSV intervention on the gut microbiota in T2DM mice. **(A)** Composition of the gut microbiota at the phylum level. **(B)** The ratio of Firmicutes/Bacteroidetes in different groups. **(C)** Composition of the gut microbiota at the genus level. **(D)** Venn diagram of the different groups based on the OTU level. **(E)** LEfSe analysis of representative gut microbes at the genus level (LDA score > 2.5, *p* < 0.05). Notes: Data are mean ± SD (*n* = 6). For **(B)**, one-way ANOVA with Tukey’s *post hoc* test was used; for **(E)**, the Kruskal–Wallis test was used, and only taxa with LDA > 2.5 and *p* < 0.05 are shown. *p < 0.05 compared with each group.

As shown in Figure 4A, the microbial composition across various sample groups is presented at the phylum level. Treatment with SV, F, and FSV results in a reduced Firmicutes/Bacteroidota (F/B) ratio in all experimental groups compared to the Model group (Figure 4B). Notably, FSV administration leads to a more pronounced reduction in the F/B ratio than F alone (*p* < 0.05). Figure 4C illustrates the microbial composition at the genus level, highlighting variations in the relative abundance of different genera. The Venn diagram in Figure 4D further emphasizes the influence of SV, F, and FSV on gut microbiota composition by showing shared and unique microbial species across the groups. Finally, Figure 4E depicts the characteristic microbial populations enriched in the gut microbiota of mice. In the Control group, healthy mice predominantly harbored *Alloprevotella* and *Bifidobacterium*. In contrast, the Model group, representing T2DM mice, exhibited significant enrichment of *Merdicola*. The SV-treated group showed a higher prevalence of *Odoribacter* and *Tidjanibacter*, while *Dwaynesavagella* became the dominant microbe in the F group. Notably, the FSV group of T2DM mice demonstrated marked enrichment of *Prevotella* and *Anaerotignum*.

A stable gut microbiota is essential for maintaining human health, while dysbiosis is linked to various diseases. This study demonstrates that SV, F, and FSV all contribute to the reconstruction and enrichment of the gut microbiota in T2DM mice, simultaneously alleviating gut damage. Comparative analysis of the F/B ratio indicates that FSV has a more profound impact on the gut microbiota of T2DM mice compared to F alone. Furthermore, FSV was found to be more effective in improving T2DM conditions. Previous studies have identified *Alloprevotella* (50), *Tidjanibacter* (50), and *Anaerotignum* (51) as beneficial probiotics, while an increase in *Prevotella* has been associated with reduced hyperglycemia in diabetic patients (52). However, whether these microbial changes directly mediate the glucose-lowering effects of FSV remains correlative and requires further functional validation.

### 3.4 Effects of SV, F, and FSV on liver metabolites in T2DM mice

**Figure 5:**
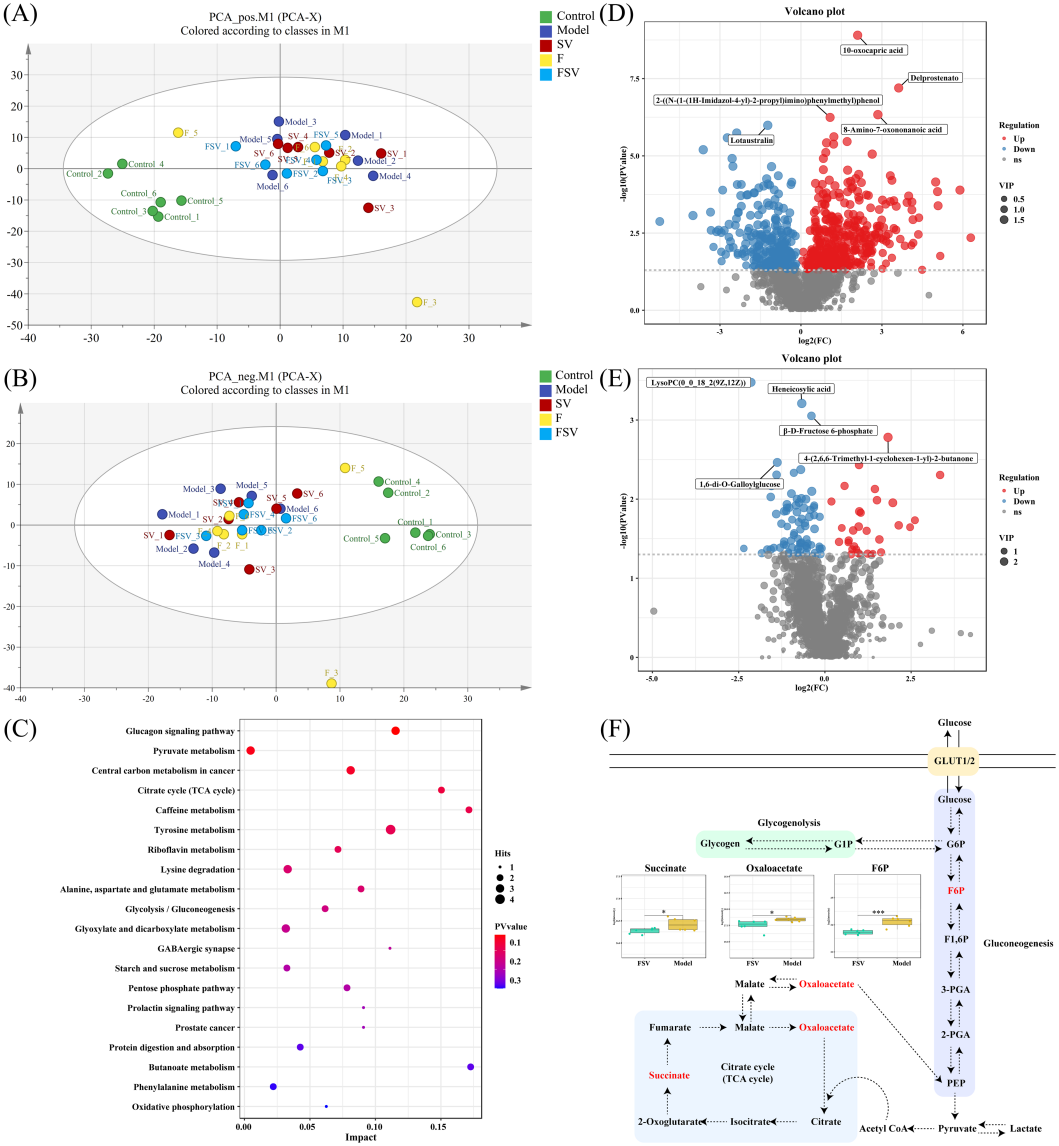
Principal component analysis (PCA) score plots of liver metabolites of different groups of mice under different modes **(A,B)**. Effects of FSV on the liver metabolites in T2DM mice: **(C)** Volcano plot between control and model group, **(D)** Volcano plot between FSV and model group, **(E)** KEGG enrichment analysis, **(F)** mechanism analysis of FSV in glucagon signaling pathway. Criteria for the screening of different metabolites: ppm ≤ 10, FDR ≤ 0.05, VIP ≥ 1, *p < 0.05, and ***p < 0.001.

Figures 5A,B present the PCA results of liver metabolites from the different groups of mice. The OPLS-DA permutation test results, shown in Supplementary Figures S4, S5, further confirm the robustness of the models, supporting their reliability in predicting outcomes. Figure 5C highlights the significant differential metabolites between the Control and Model groups (*p* < 0.05). Figure 5D presents the significant differential metabolites identified when comparing the Model and FSV groups (*p* < 0.05). Among the notable differential metabolites in the livers of Control and Model group T2DM mice, 10-oxocapric acid, delprostenato, and 8-amino-7-oxononanoic acid were significantly upregulated, while lotaustralin showed marked downregulation. In contrast, when comparing the Model and FSV groups, 4-(2,6,6-trimethyl-1-cyclohexen-1-yl)-2-butanone was significantly upregulated, while lysoPC(0_0_18_2(9Z,12Z)), heneicosylic acid, β-D-fructose 6-phosphate (F6P), and 1,6-di-O-galloylglucose were significantly downregulated. KEGG enrichment analysis of the significant differential metabolites between the Model and FSV groups identified several potential metabolic pathways (Figure 5E). Notably, only three signaling pathways showed significant differences: the glucagon signaling pathway (Supplementary Figure S6), pyruvate metabolism, and central carbon metabolism in cancer. The glucagon signaling pathway was the most prominent, as shown in Figure 5F, revealing that T2DM significantly elevated the levels of three metabolites: F6P, oxaloacetate, and succinic acid (*p* < 0.05).

Although network pharmacology analysis identified multiple pathways—such as neuroactive ligand-receptor interaction, the PI3K-Akt signaling pathway, and actin cytoskeleton regulation—through which FSV may modulate T2DM, metabolomics analysis pinpointed the glucagon signaling pathway as the primary mechanism. Notably, FSV significantly reduced the levels of three key metabolites within this pathway: F6P, oxaloacetate, and succinic acid (*p* < 0.05). As illustrated in Figure 5F, F6P, an intermediate in glycolysis and gluconeogenesis, is elevated in T2DM, suggesting enhanced gluconeogenesis and suppressed glycolysis, thereby redirecting carbon sources toward glucose synthesis (53). Increased oxaloacetate levels indicate that the liver is utilizing lactate from muscle and adipose tissue more efficiently to synthesize oxaloacetate, thereby supporting gluconeogenesis (54). Succinic acid, a pro-inflammatory signaling molecule and key intermediate in the TCA cycle (55), accumulates as both a marker of liver metabolic dysfunction and a contributor to insulin resistance and hepatocyte injury (56), consistent with our findings. The concurrent elevation of these metabolites suggests that, due to insulin resistance, T2DM mice cannot effectively utilize glucose. This dysregulated signaling leads the liver to perceive an energy deficit, resulting in excessive glucose synthesis. The uncontrolled glucagon signaling pathway thus contributes to hyperglycemia and disrupted glucose homeostasis in T2DM mice. Intervention with FSV was associated with attenuation of the glucagon signaling pathway dysregulation, as reflected by changes in the levels of F6P, oxaloacetate, and succinic acid. From a metabolomics perspective, FSV represents a promising functional food for T2DM intervention and generates hypotheses for future research into the specific targets through which FSV may regulate this condition.

### 3.5 Non-targeted metabolomics screening combined with network pharmacology reveals the potential mechanism of FSV in regulating T2DM

**Figure 6:**
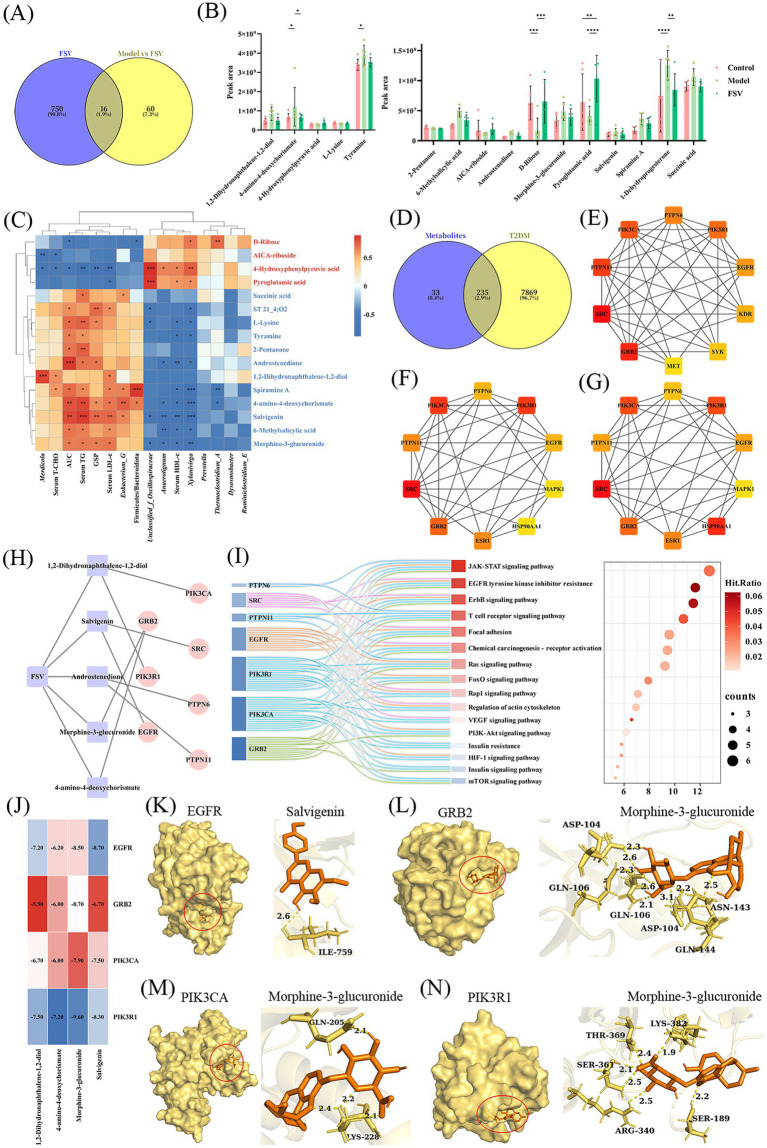
Mechanism of FSV in regulating T2DM based on metabolomics and network pharmacology. **(A)** Venn diagram of FSV components and differential metabolites (between the model and FSV groups of T2DM mice), **(B)** content changes of differential metabolites in the liver of mice from the control, model, and FSV groups, **(C)** correlation heatmap between differential metabolites and physiological indicators, **(D)** Venn diagram of potential targets of differential metabolites and T2DM-related targets, **(E)** top 10 genes in the intersection targets based on the MCC, **(F)** top 10 genes in the intersection targets based on the MNC, **(G)** top 10 genes in the intersection targets based on the degree, **(H)** network diagram of shared targets from **(E–G)** and their corresponding metabolites, **(I)** Sankey diagram of shared targets and T2DM-related signaling pathways, **(J)** Heatmap of minimum binding energy between core targets and metabolites (kcal/mol), **(K)** Visualization of molecular docking between EGFR and salvigenin, **(L)** Visualization of molecular docking between GRB2 and M3G, **(M)** Visualization of molecular docking between PIK3CA and M3G, **(N)** Visualization of molecular docking between PIK3R1 and M3G.

To investigate the mechanisms by which FSV regulates T2DM, this study performed an intersection analysis of metabolites present in FSV and those identified in the livers of Model and FSV groups of T2DM mice. The analysis revealed 16 shared metabolites (Figure 6a), including 1,2-dihydronaphthalene-1,2-diol, 4-amino-4-deoxychorismate, and tyramine (Supplementary Table S3). The changes in the relative concentrations of these metabolites across the Control, Model, and FSV groups are illustrated in Figure 6b. Following FSV intervention, four metabolites showed increased levels, while 12 exhibited decreased levels, with the specific trends outlined in Supplementary Table S3. Based on these metabolite alterations, correlation analyses were performed to explore the relationships between the differential metabolites and physiological indicators (Figure 6c). The analysis revealed that 4-hydroxyphenylpyruvic acid negatively correlated with *Merdicola*, serum T-CHO, and AUC (*p* < 0.05). Spiramine A was negatively correlated with *Xylanivirga* and serum HDL-c (*p* < 0.05), while 4-amino-4-deoxychorismate positively correlated with serum TG, AUC, and *Eubacterium_G* (*p* < 0.01). These correlations suggest that these metabolites play a role in FSV’s intervention in T2DM by regulating glucose and lipid metabolism, as well as gut microbiota composition. To predict the underlying molecular mechanisms, network pharmacology analysis was used to explore the relationships between the 16 differential metabolites and T2DM. Intersection analysis (Figure 6d) identified 235 common targets shared between the metabolites and T2DM-related targets. A PPI network was constructed for these 235 targets, and core targets were filtered using three algorithms—MCC, MNC, and Degree—via the cytoHubba tool in Cytoscape software (Figures 6e–g). Seven core targets were identified across all three algorithms: GRB2, SRC, PTPN11, PIK3CA, PTPN6, PIK3R1, and EGFR. The relationships between these core targets and their corresponding metabolites were visualized in an interaction network (Figure 6h). To further elucidate the pathways associated with these core targets, a Sankey diagram linking the seven targets to T2DM-related pathways was constructed (Figure 6i). The analysis revealed that EGFR, PIK3R1, PIK3CA, and GRB2 had the highest associations with these pathways, suggesting their pivotal role in improving T2DM. Molecular docking simulations were performed to validate the binding activity of the core targets with the metabolites. The lowest binding energy results are presented in Figure 6j, with visualized docking patterns shown in Figures 6k–n. These findings suggest that morphine-3-glucuronide (M3G) is a central metabolite whose levels changed following FSV intervention. Molecular docking predicted low binding energies between M3G and GRB2, PIK3CA, and PIK3R1, with M3G positioned within the active pockets of these proteins. These *in silico* predictions raise the hypothesis that M3G might interact with these targets, but this requires experimental testing.

A phenomenon was observed wherein certain metabolites showed decreased relative concentrations following FSV intervention, despite their established positive regulatory effects on T2DM. This may be attributed to FSV’s capacity to repair the gut and enhance intestinal permeability, thereby increasing the utilization of these metabolites within the gut. As a result, only a fraction of these metabolites enter the bloodstream and accumulate in the liver. FSV intervention modulates the relative concentrations of 16 hepatic metabolites, which are significantly associated with blood glucose levels and gut microbiota, suggesting that these metabolites serve as key mediators of FSV’s effects on T2DM. Among these metabolites, 4-hydroxyphenylpyruvic acid is recognized for its anti-diabetic properties, primarily through inhibition of *α*-glucosidase (57). Salvigenin offers protective effects on liver tissue through antioxidant and anti-inflammatory mechanisms (58). Tyramine is believed to exert anti-diabetic effects by promoting insulin secretion and enhancing insulin sensitivity (59).

Interestingly, M3G showed low predicted binding energies with PIK3CA, PIK3R1, and EGFR in our docking analysis, suggesting a potential interaction that warrants experimental validation. Although classical opioid signaling is well-documented, the accumulation of endogenous M3G in the diabetic liver may represent a form of “metabolic toxicity,” a hypothesis supported by our correlative data. Unlike morphine-6-glucuronide, M3G lacks analgesic effects and has neuro-excitatory properties. Research indicates that M3G accumulates in T2DM and is difficult to eliminate (60). Once in circulation, M3G can significantly elevate serum glucose levels in rats (61). This accumulation could be tentatively interpreted as a “Compensation Paradox,” wherein metabolic stress triggers endogenous morphine synthesis while impaired hepatic glucuronidation or transport results in M3G retention – a conceptual framework to guide future studies.

**Figure 7:**
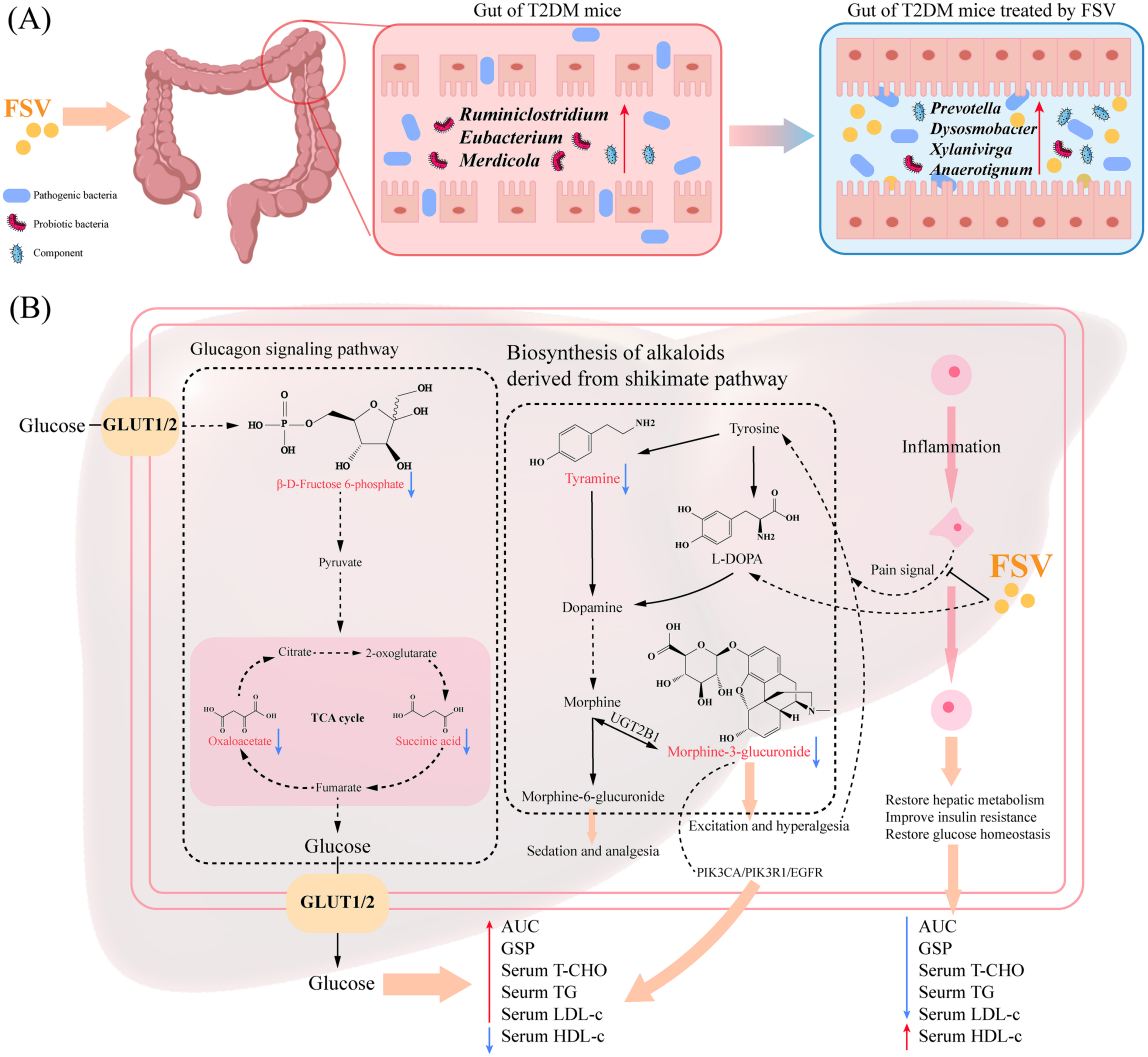
Hypothesis on the mechanism of FSV in regulating T2DM. **(A)** Mechanism of FSV in regulating gut microbiota in T2DM mice; **(B)** Mechanism of FSV in improving hepatic function in T2DM mice.

Based on existing research and our correlative findings, this study proposes a hypothetical integrated mechanism for M3G’s role in T2DM (Figure 7) that requires direct experimental testing. Based on the literature (62) rather than direct measurements in our study, the chronic stress associated with T2DM may hypothetically trigger compensatory activation of the tyrosine-dopamine-endogenous morphine axis, a speculative model that awaits biochemical validation. However, this compensation may hypothetically encounter pathological hepatic alterations: upregulation of UGT2B1 could accelerate the conversion of morphine to M3G, while abnormal expression of MRP2/3 might impede M3G excretion, together leading to its accumulation and creating a compensation paradox. Based on a previous report and our *in silico* results, accumulated M3G might hypothetically contribute to metabolic dysregulation through two main pathways: firstly, via direct central nervous system stimulation leading to hyperglycemia and increased stress hormone release (63); secondly, as suggested by our molecular docking, through possible interactions with targets such as PIK3CA/PIK3R1 and EGFR, which could hypothetically interfere with insulin signaling. These possibilities are speculative and await experimental confirmation. This could initiate a putative vicious cycle of “metabolic dysregulation → increased M3G production → worsening dysregulation,” a hypothesis that now requires direct experimental interrogation. Our findings indicate that after FSV intervention, levels of metabolites such as tyramine and M3G decrease, which is an observational result rather than a proven causal effect. This effect may be attributable, at least speculatively, to FSV’s role in repairing the intestinal barrier, which could promote the efflux of exogenous M3G from intestinal epithelial cells and reduce its absorption, but this mechanism remains to be directly validated. Additionally, overall metabolic improvement might reduce the need for endogenous morphine, thereby potentially lowering tyrosine consumption and M3G production, though this inference is based on correlation rather than causation. If confirmed in the future studies, this hypothesized dual action might offer a new perspective on neuroendocrine-metabolic interactions in T2DM.

Several limitations of this study should be acknowledged. First, the molecular docking results suggesting interactions between M3G and PIK3CA/PIK3R1/EGFR are purely computational and await experimental validation using techniques such as surface plasmon resonance or cellular thermal shift assays. Second, the proposed hypothesis involving the “tyrosine-dopamine-endogenous morphine axis” and the “compensation paradox” is speculative, as it is based on literature correlation rather than direct biochemical measurements in our mouse model. These limitations do not detract from the main conclusions but should be considered when interpreting the findings. Additionally, *a priori* power calculation was not performed before the experiment; the sample size was chosen based on previous similar studies. Although *post hoc* power analysis indicated that the sample size was adequate for the primary endpoints, we acknowledge that a priori calculation would have provided stronger justification.

## 4 Conclusion

This study elucidated the bioactive profile of FSV and identified a potential metabolic correlate of its anti-diabetic effects, warranting further mechanistic investigation. Through a combination of chemical profiling and network pharmacology, flavonoids (e.g., apigenin, tangeritin) were identified as the potential bioactive constituents of FSV. Further integration of metabolomics and computational biology revealed that M3G is a hepatic metabolite whose relative abundance was lower in FSV-treated mice compared to the Model group. *In vivo* experiments showed that FSV significantly improved hyperglycemia and hepatic steatosis in T2DM mice. Notably, FSV was associated with reduced hepatic M3G levels, and molecular docking raised the hypothesis that M3G might interact with components of the PI3K-Akt signaling pathway; however, this possibility has not been directly tested and requires experimental validation. These findings suggest that FSV may influence the hepatic metabolome, and provide preliminary, correlational evidence that links endogenous M3G regulation to diabetes management.

## Data Availability

The datasets presented in this study can be found in online repositories. The names of the repository/repositories and accession number(s) can be found at: PRJNA1434130.

## Footnotes

1
http://www.massbank.jp/

2
http://www.lipidmaps.org

3
https://www.mzcloud.org

4
https://www.omim.org/

## References

[1] Zhang L Ai C Guo C Li S Niu J Meng X . Ucp2 inhibition exaggerates diabetic cardiomyopathy by facilitating the activation of nlrp3 and pyroptosis. Diabetol Metab Syndr. (2025) 17:267. doi: 10.1186/s13098-025-01855-w, 40671041 PMC12269110

[2] Zhang K Qi Y Wang W Tian X Wang J Xu L . Future horizons in diabetes: integrating ai and personalized care. Front Endocrinol (Lausanne). (2025) 16:1583227. doi: 10.3389/fendo.2025.1583227, 40213102 PMC11983400

[3] Mohamed HA Mohamed NA Macasa SS Basha HK Adan AM Crovella S . Metformin-loaded nanoparticles reduce hyperglycemia-associated oxidative stress and induce enos phosphorylation in vascular endothelial cells. Sci Rep. (2024) 14:30870. doi: 10.1038/s41598-024-81427-6, 39730492 PMC11681025

[4] Xie S Liu M Kong Y Yang Y Chen R Wang Y . Mesenchymal stromal/stem cell-derived exosomes as a potential therapeutic approach to osteoarthritis combined with type 2 diabetes mellitus. Front Cell Dev Biol. (2025) 13:1549096. doi: 10.3389/fcell.2025.1549096, 40213388 PMC11983606

[5] Peres NDSL Bortoluzzi LCP Cardoso FAR Fuchs RHB Brito NLH Trelha SG . Partial replacement of cassive starch with peruvian maca flour in mortadella: innovation in functional meat products with clea-label potential. J Food Sci. (2025) 90:e70314. doi: 10.1111/1750-3841.70314, 40476759 PMC12143192

[6] Martínez R García-Beltrán A Kapravelou G Mesas C Cabeza L Perazzoli G . *In vivo* nutritional assessment of the microalga *Nannochloropsis gaditana* and evaluation of the antioxidant and antiproliferative capacity of its functional extracts. Mar Drugs. (2022) 20:318. doi: 10.3390/md20050318, 35621969 PMC9147351

[7] Yao X Lu X Cao D Huang L Zhou Z Qiu Z . Functional food ge-zhi soup ameliorates acute liver injury through the akt/gsk3β/pparα pathway. Food Sci Nutr. (2025) 13:e70603. doi: 10.1002/fsn3.70603, 40697704 PMC12280231

[8] Li H Zheng J Wu Y Zhou H Zeng S Li Q. *Dendrobium officinale* polysaccharide decreases podocyte injury in diabetic nephropathy by regulating irs-1/akt signal and promoting mitophagy. Aging. (2023) 15:10291–306. doi: 10.18632/aging.205075, 37812195 PMC10599763

[9] Zhang Y Wang T Han J Song J Yang C Liang L . Targeting the gut microbiota and lipid metabolism: potential mechanisms of natural products for the treatment of non-alcoholic fatty liver disease. Front Pharmacol. (2025) 16:1610498. doi: 10.3389/fphar.2025.1610498, 40552158 PMC12183287

[10] Ferenczyova K Kalocayova B Bartekova M. Potential implications of quercetin and its derivatives in cardioprotection. Int J Mol Sci. (2020) 21:1585. doi: 10.3390/ijms21051585, 32111033 PMC7084176

[11] Wang F Li Y Zhang Y Zhou Y Li S Li H. Natural products for the prevention and treatment of hangover and alcohol use disorder. Molecules. (2016) 21:64. doi: 10.3390/molecules21010064, 26751438 PMC6274469

[12] Luo Z Zhou W Xie T Xu W Shi C Xiao Z . The role of botanical triterpenoids and steroids in bile acid metabolism, transport, and signaling: pharmacological and toxicological implications. Acta Pharm Sin B. (2024) 14:3385–415. doi: 10.1016/j.apsb.2024.04.027, 39220868 PMC11365449

[13] Shi K Jing B Feng Y Yu Y. *Anemarrhena asphodeloides* bunge total saponins lower lipid via modulating maoa activity to enhance defense mechanisms in mice and c. elegans. J Ethnopharmacol. (2025) 337:118814. doi: 10.1016/j.jep.2024.118814, 39277062

[14] Zhou X Li Y Yang Y Wei L Wang C Xu J . Regulatory effects of *poria cocos* polysaccharides on gut microbiota and metabolites: evaluation of prebiotic potential. Npj Sci Food. (2025) 9:53. doi: 10.1038/s41538-025-00416-9, 40263347 PMC12015419

[15] Zhao D Wang Y Wu S Ji X Gong K Zheng H . Research progress on the role of macrophages in acne and regulation by natural plant products. Front Immunol. (2024) 15:1383263. doi: 10.3389/fimmu.2024.1383263, 38736879 PMC11082307

[16] Komolafe K Olaleye MT Huang H Pacurari M. Contemporary insights into the biological mechanisms of *Parkia biglobosa*. Int J Environ Res Public Health. (2024) 21:394. doi: 10.3390/ijerph21040394, 38673307 PMC11050164

[17] Coşkuner G Alkay Z Arioglu-Tuncil S Gonzales MAA Lindemann SR Tunçil YE. In vitro fecal microbiota modulation properties of some dried apricots (*Prunus armeniaca* l.). J Sci Food Agric. (2025) 105:7429–39. doi: 10.1002/jsfa.70003, 40519172

[18] Ping Y Liu J Wang H Wang Y Qiu H Zhang Y. Research progress in the treatment of an immune system disease-type 1 diabetes-by regulating the intestinal flora with chinese medicine and food homologous drugs. Biosci Microbiota Food Health. (2024) 43:150–61. doi: 10.12938/bmfh.2023-068, 38966054 PMC11220337

[19] Huang H Li X Lu Q Xu H Sun H Zhang J . Optimized liquid medium formulation for *Sanghuangporus vaninii* and biological activity of the exopolysaccharides. Foods. (2024) 13:3574. doi: 10.3390/foods13223574, 39593990 PMC11593129

[20] Ding D Wang H Liang J Su C Zhang M Jiao F . Genomic analysis of *Inonotus hispidus* provides insights into its medicinal properties and evolutionary dynamics. Sci Rep. (2025) 15:23031. doi: 10.1038/s41598-025-05528-6, 40594532 PMC12215927

[21] Jiang R Cong Z Zheng L Zhang L Guan Q Wang S . Global research trends in regulating gut microbiome to improve type 2 diabetes mellitus: bibliometrics and visual analysis. Front Endocrinol (Lausanne). (2024) 15:1401070. doi: 10.3389/fendo.2024.1401070, 38887274 PMC11181692

[22] Nguyen PC Nguyen MTT Truong BT Kim D Shin S Kim J . Isolation, physicochemical characterization, and biological properties of inotodiol, the potent pharmaceutical oxysterol from chaga mushroom. Antioxidants (Basel). (2023) 12:447. doi: 10.3390/antiox12020447, 36830005 PMC9952744

[23] Huang Z Chen J Wang C Xiao M Zhu Y Li N . Antidiabetic potential of *Chlorellapyrenoidosa* functional formulations in streptozocin-induced type 2 diabetic mice. J Funct Foods. (2023) 103:105489. doi: 10.1016/j.jff.2023.105489

[24] Dudášová S Wurz J Berger U Reemtsma T Fu Q Lechtenfeld OJ. An automated and high-throughput data processing workflow for pfas identification in biota by direct infusion ultra-high resolution mass spectrometry. Anal Bioanal Chem. (2024) 416:4833–48. doi: 10.1007/s00216-024-05426-2, 39090266 PMC11330400

[25] Wishart DS Guo A Oler E Wang F Anjum A Peters H . HMDB 5.0: the human metabolome database for 2022. Nucleic Acids Res. (2022) 50:D622–31. doi: 10.1093/nar/gkab1062, 34986597 PMC8728138

[26] Kanehisa M Furumichi M Sato Y Matsuura Y Ishiguro-Watanabe M. KEGG: biological systems database as a model of the real world. Nucleic Acids Res. (2025) 53:D672–7. doi: 10.1093/nar/gkae909, 39417505 PMC11701520

[27] Kim S Chen J Cheng T Gindulyte A He J He S . Pubchem 2025 update. Nucleic Acids Res. (2024) 53:D1516–25. doi: 10.1093/nar/gkae1059, 39558165 PMC11701573

[28] Daina A Michielin O Zoete V. SwissADME: a free web tool to evaluate pharmacokinetics, drug-likeness and medicinal chemistry friendliness of small molecules. Sci Rep. (2017) 7:42717. doi: 10.1038/srep42717, 28256516 PMC5335600

[29] Daina A Zoete V. Testing the predictive power of reverse screening to infer drug targets, with the help of machine learning. Commun Chem. (2024) 7:105. doi: 10.1038/s42004-024-01179-2, 38724725 PMC11082207

[30] Stelzer G Rosen N Plaschkes I Zimmerman S Twik M Fishilevich S . The genecards suite: from gene data mining to disease genome sequence analyses. Curr Protoc Bioinformatics. (2016) 54:1.30.1–1.30.33. doi: 10.1002/cpbi.5, 27322403

[31] Knox C Wilson M Klinger CM Franklin M Oler E Wilson A . Drugbank 6.0: the drugbank knowledgebase for 2024. Nucleic Acids Res. (2024) 52:D1265–75. doi: 10.1093/nar/gkad976, 37953279 PMC10767804

[32] Nagam V. Early detection of temporal lobe epilepsy: identification of novel candidate genes and potential biomarkers using integrative genomics analysis. Open J Genet. (2020) 10:65–81. doi: 10.4236/ojgen.2020.104006

[33] Bader GD Hogue CWV. An automated method for finding molecular complexes in large protein interaction networks. BMC Bioinformat. (2003) 4:2. doi: 10.1186/1471-2105-4-2, 12525261 PMC149346

[34] Nie H Liang C He Y Wang S Zhang X Duan J . Implant renal injury-responsive cells to supplement erythropoietin and protect kidney injury. MedComm. (2025) 6:e70438. doi: 10.1002/mco2.70438, 41179707 PMC12572954

[35] Li Z Huang Z Luo Y Cheng W Liu Y Zhong Y . *Gracilaria* extract reduce hyperglycemia by modulating gut microbial and short chain fatty acids. J Agric Food Res. (2024) 18:101100. doi: 10.1016/j.jafr.2024.101100

[36] Zelena E Dunn WB Broadhurst D Francis-McIntyre S Carroll KM Begley P . Development of a robust and repeatable uplc-ms method for the long-term metabolomic study of human serum. Anal Chem. (2009) 81:1357–64. doi: 10.1021/ac8019366 19170513

[37] Want EJ Masson P Michopoulos F Wilson ID Theodoridis G Plumb RS . Global metabolic profiling of animal and human tissues via uplc-ms. Nat Protoc. (2013) 8:17–32. doi: 10.1038/nprot.2012.135, 23222455

[38] Chen C Wu Y Li J Wang X Zeng Z Xu J . Tbtools-ii: a “one for all, all for one” bioinformatics platform for biological big-data mining. Mol Plant. (2023) 16:1733–42. doi: 10.1016/j.molp.2023.09.010, 37740491

[39] Liu H Fan Y Liao H Liu Y Chen S Ma Z . Apigenin alleviates stz-induced diabetic cardiomyopathy. Mol Cell Biochem. (2017) 428:9–21. doi: 10.1007/s11010-016-2913-9, 28176247

[40] Fikry E Orfali R El-Sayed SS Perveen S Ghafar S El-Shafae AM . Potential hepatoprotective effects of *Chamaecyparis lawsoniana* methotrexate-induced liver injuty: integrated phytochemical profiling, target network analysis, and experimental validation. Antioxidants. (2023) 12:2118. doi: 10.3390/antiox12122118, 38136237 PMC10740566

[41] Xiao D Su X Gao H Li X Qu Y. The roles of lpar1 in central nervous system disorders and diseases. Front Neurosci. (2021) 15:710473. doi: 10.3389/fnins.2021.710473, 34385905 PMC8353257

[42] Qi Y Ni S Heng X Qu S Ge P Zhao X . Uncovering the potential mechanisms of *coptis chinensis* franch. For serious mental illness by network pharmacology and pharmacology-based analysis. Drug Des Devel Ther. (2022) 16:325–42. doi: 10.2147/DDDT.S342028, 35173416 PMC8841750

[43] Ye C Li Y Shi J He L Shi X Yang W . Network pharmacology analysis revealed the mechanism and active compounds of jiao tai wan in the treatment of type 2 diabetes mellitus via src/pi3k/akt signaling. J Ethnopharmacol. (2025) 337:118898. doi: 10.1016/j.jep.2024.118898, 39374878

[44] Liu Z Zhang Z Chen X Ma P Peng Y Li X. Citrate and hydroxycinnamate derivatives from mume fructus protect lps-injured intestinal epithelial cells by regulating the fak/pi3k/akt signaling pathway. J Ethnopharmacol. (2023) 301:115834. doi: 10.1016/j.jep.2022.115834, 36270558

[45] Liang Y Li L Liang J Liu D Chu S Li H. Integrating mendelian randomization and single-cell RNA sequencing to identify therapeutic targets of baicalin for type 2 diabetes mellitus. Front Pharmacol. (2024) 15:1403943. doi: 10.3389/fphar.2024.1403943, 39130628 PMC11310057

[46] Chen L Wei C. Integrative bioinformatics and experimental study of calcium metabolism-related genes in gestational diabetes mellitus. Medicine. (2025) 104:e45334. doi: 10.1097/MD.0000000000045334, 41137247 PMC12558329

[47] Velmurugan S Despa S. Calcium signaling and cardiac adaptation to stress: focus on pregnancy and diabetes. Biomolecules. (2025) 15:1421. doi: 10.3390/biom15101421, 41154650 PMC12564852

[48] Wang M Cai H Wang M Rong R Zhang J Zhang Z . Lonicerin targets adra1d and rspo3 to ameliorate diabetes-induced vascular injury through ca2+/calcineurin/nfat1-dependent anti-endmt pathway. Phytomedicine. (2025) 143:156832. doi: 10.1016/j.phymed.2025.156832, 40398181

[49] Dai B Wu Q Zeng C Zhang J Cao L Xiao Z . The effect of Liuwei Dihuang decoction on Pi3k/Akt signaling pathway in liver of type 2 diabetes mellitus (T2DM) rats with insulin resistance. J Ethnopharmacol. (2016) 192:382–9. doi: 10.1016/j.jep.2016.07.024, 27401286

[50] Chen Z Ge X Wang Y Zhang J Sui Y Yin X . *Ruditapesphilippinarum* polysaccharide alleviates hyperglycemia by modulating gut microbiota in a mouse model of type 2 diabetes mellitus. Mol Nutr Food Res. (2025) 69:e202400996. doi: 10.1002/mnfr.202400996, 39981981

[51] Hata S Okamura T Kobayashi A Bamba R Miyoshi T Nakajima H . Gut microbiota changes by an SGLT2 inhibitor, luseogliflozin, alters metabolites compared with those in a low carbohydrate diet in db/db mice. Nutrients. (2022) 14:3531. doi: 10.3390/nu14173531, 36079789 PMC9459736

[52] Wang P Hu C Li Q Wu K Chai X Fu X . *Fructus mori* polysaccharides modulate the axial distribution of gut microbiota and fecal metabolites to improve symptoms of hyperglycemia in type 2 diabetic mice. Int J Biol Macromol. (2025) 307:141949. doi: 10.1016/j.ijbiomac.2025.141949 40074112

[53] Dewdney B Roberts A Qiao L George J Hebbard L. A sweet connection? Fructose's role in hepatocellular carcinoma. Biomolecules. (2020) 10:496. doi: 10.3390/biom10040496, 32218179 PMC7226025

[54] Pendleton AL Wesolowski SR Regnault TRH Lynch RM Limesand SW. Dimming the powerhouse: mitochondrial dysfunction in the liver and skeletal muscle of intrauterine growth restricted fetuses. Front Endocrinol. (2021) 12:612888. doi: 10.3389/fendo.2021.612888, 34079518 PMC8165279

[55] Zheng Y Xu R Chen X Lu Y Zheng J Lin Y . Metabolic gatekeepers: harnessing tumor-derived metabolites to optimize t cell-based immunotherapy efficacy in the tumor microenvironment. Cell Death Dis. (2024) 15:775. doi: 10.1038/s41419-024-07122-6, 39461979 PMC11513100

[56] Hosomi K Saito M Park J Murakami H Shibata N Ando M . Oral administration of *blautia wexlerae* ameliorates obesity and type 2 diabetes via metabolic remodeling of the gut microbiota. Nat Commun. (2022) 13:4477. doi: 10.1038/s41467-022-32015-7, 35982037 PMC9388534

[57] Nipun TS Khatib A Ibrahim Z Ahmed QU Redzwan IE Primaharinastiti R . Gc-ms- and nmr-based metabolomics and molecular docking reveal the potential alpha-glucosidase inhibitors from *Psychotria malayana* jack leaves. Pharmaceuticals (Basel). (2021) 14:978. doi: 10.3390/ph14100978, 34681203 PMC8541227

[58] Edwards H Javed K Yadev K Ara C Omer A. Therapeutic potential of salvigenin to combat atrazine induced liver toxicity in rats via regulating Nrf-2/Keap-1 and Nf-κb pathway. Pestic Biochem Physiol. (2024) 202:105966. doi: 10.1016/j.pestbp.2024.105966, 38879343

[59] Al-Taie A Al-Rashid AAD Rahim NN. Potential impact of tyrosine kinase inhibitors on glycemic control and diabetes mellitus progression: a clinical appraisal. J Diabetes Metab Disord. (2025) 24:291. doi: 10.1007/s40200-025-01811-5, 41393197 PMC12698884

[60] Hasegawa Y Kishimoto S Shibatani N Nomura H Ishii Y Onishi M . The pharmacokinetics of morphine and its glucuronide conjugate in a rat model of streptozotocin-induced diabetes and the expression of Mrp2, Mrp3 and Ugt2b1 in the liver. J Pharm Pharmacol. (2010) 62:310–4. doi: 10.1211/jpp.62.03.0004, 20487213

[61] Hashiguchi Y Molina PE Abumrad NN. Morphine-3-glucuronide: hyperglycemic and neuroendocrine potentiating effects. Brain Res. (1995) 694:13–20. doi: 10.1016/0006-8993(95)00697-o, 8974636

[62] Neri C Ghelardini C Sotak B Palmiter RD Guarna M Stefano G . Dopamine is necessary to endogenous morphine formation in mammalian brain *in vivo*. J Neurochem. (2008) 106:2337–44. doi: 10.1111/j.1471-4159.2008.05572.x, 18643791

[63] Raz I Hasdai D Seltzer Z Melmed RN. Effect of hyperglycemia on pain perception and on efficacy of morphine analgesia in rats. Diabetes. (1988) 37:1253–9. doi: 10.2337/diab.37.9.1253, 3410166

