# Supplementary material for: Integrated metabolomics and computational analysis suggest that a Sanghuangporus vaninii-based formulation alleviates T2DM in mice and modulates hepatic morphine-3-glucuronide axis
Source: Front Nutr. 2026 May 8;13:1826245. doi: 10.3389/fnut.2026.1826245 (PMC13194446; doi:10.3389/fnut.2026.1826245)
Supplement: Supplementary file 1 [file Supplementary_file_1.DOCX]

Supplementary Material

# Supplementary Figures and Tables

## Supplementary Figures

##
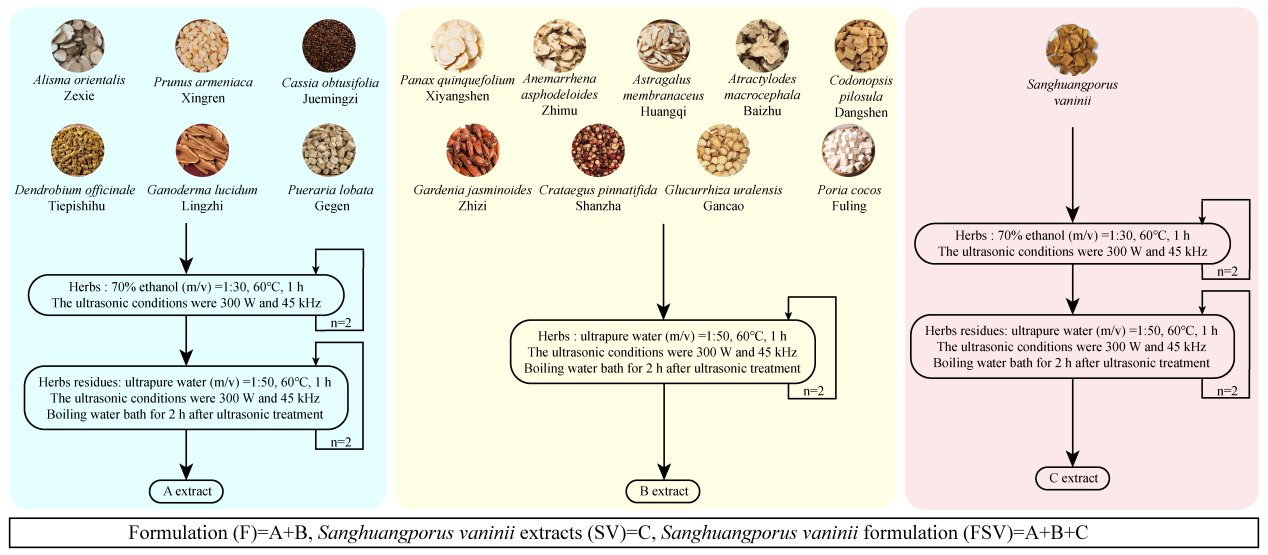
Supplementary Figure 1. Flow chart of the preparation process of formulation (F), *Sanghuangporus vaninii* extracts (SV), and *S. vaninii* formulation (FSV).


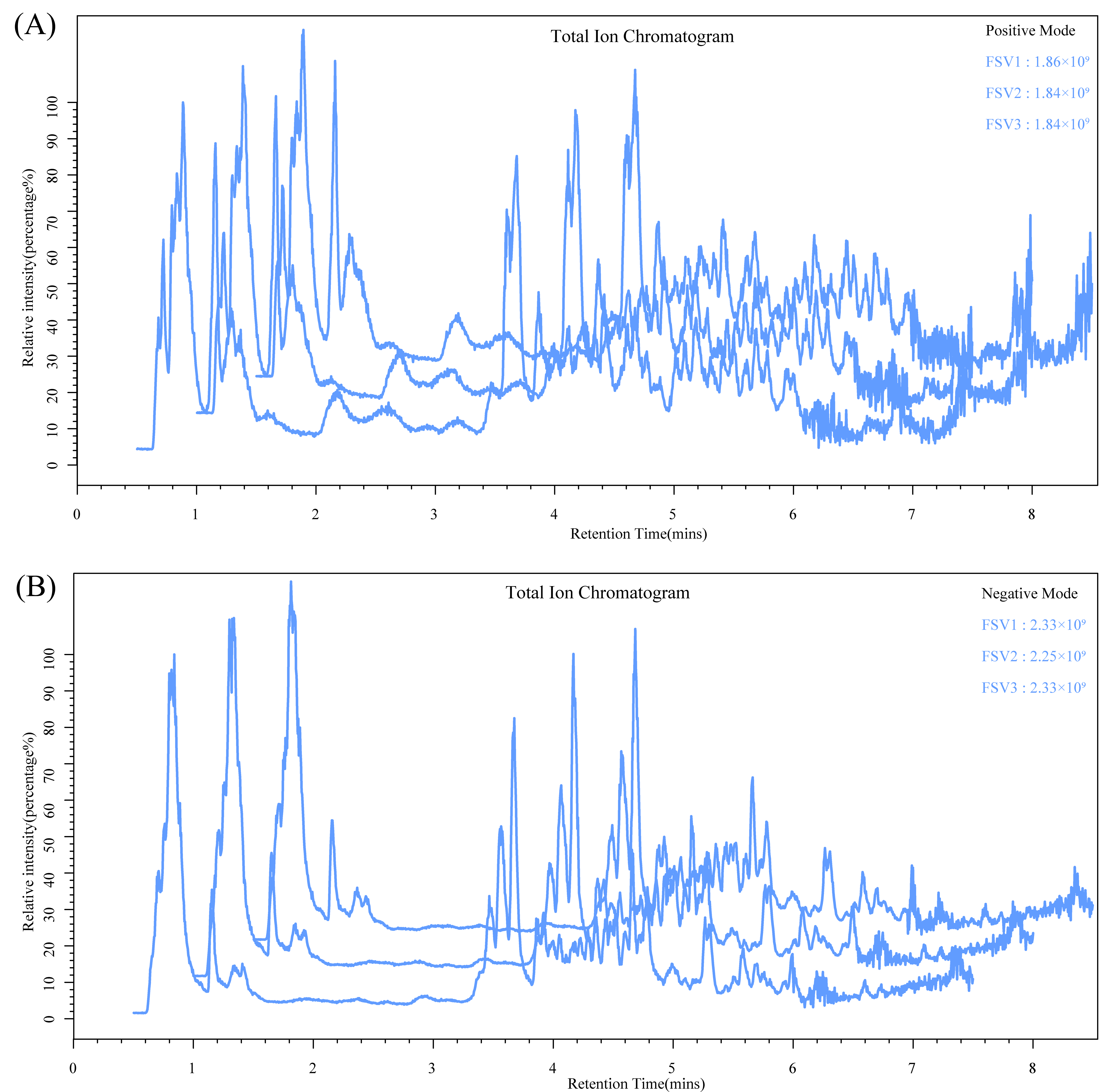


**Supplementary Figure 2.** Total ion chromatogram (TIC) of FSV under (a) positive and (b) negative ion mode.


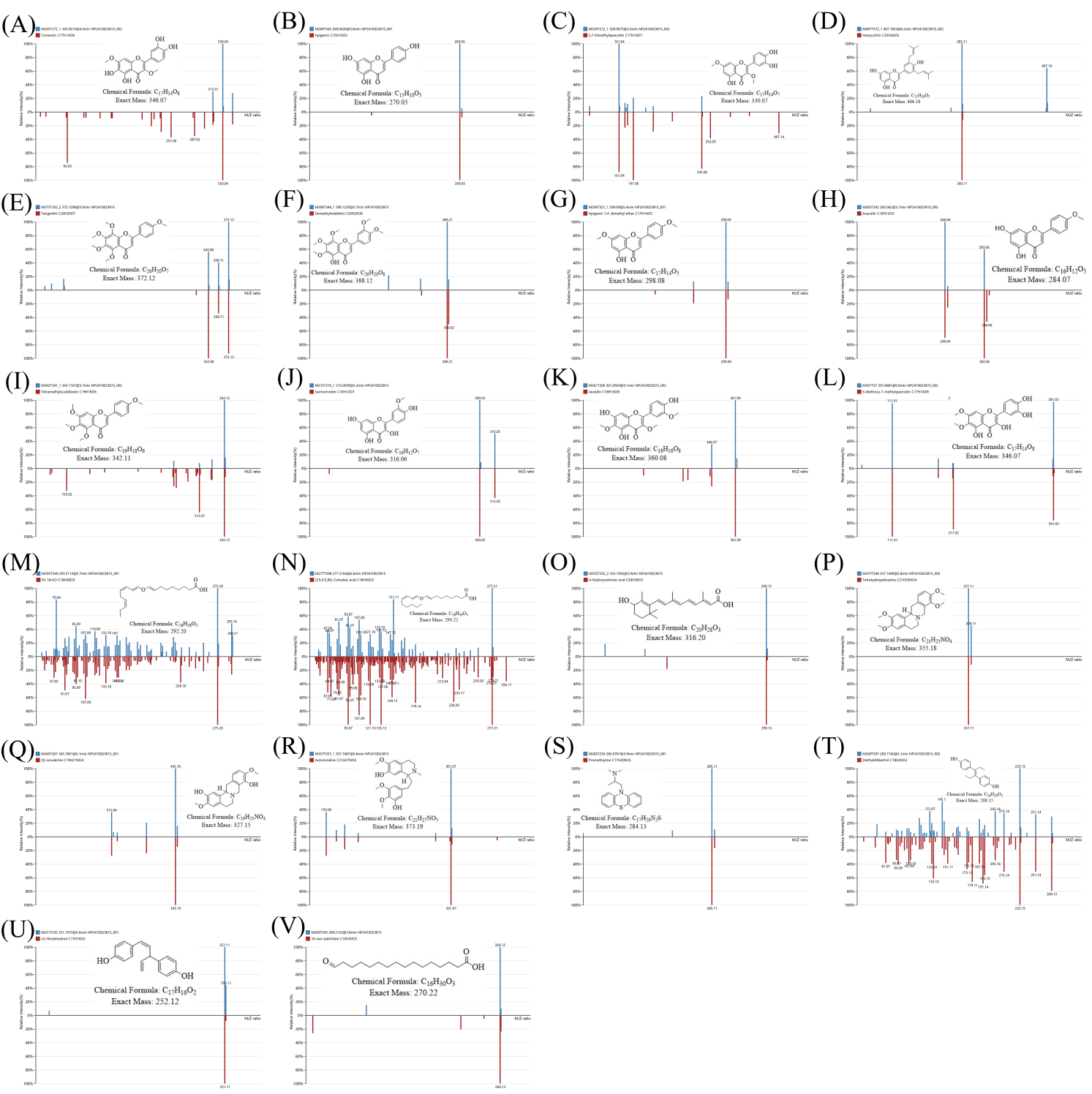


## Supplementary Figure 3. Identification plot of main compounds in FSV. (A) Tomentin; (B) Apigenin; (C) 3,7-Dimethylquercetin; (D) Honyucitrin; (E) Tangeritin; (F) Demethylnobiletin; (G) Apigenin 7,4’-dimethyl ether; (H) Acacetin; (I) Tetramethylscutellarein; (J) Isorhamnetin; (K) Jaceidin; (L) 6-Methoxy-7-methylquercetin; (M) FA 18_4;O; (N) (2’E,4’Z,8E)-Colneleic acid; (O) 4-Hydroxyretinoic acid; (P) Tetrahydropalmatine; (Q) (S)-scoulerine; (R) Autumnaline; (S) Promethazine; (T) Diethylstilbestrol; (U) cis-Hinokiresinol; (V) 16-oxo-palmitate. Notes: Each figure presents a comparison between the mass spectrum of a certain substance in FSV and that of the standard substance.


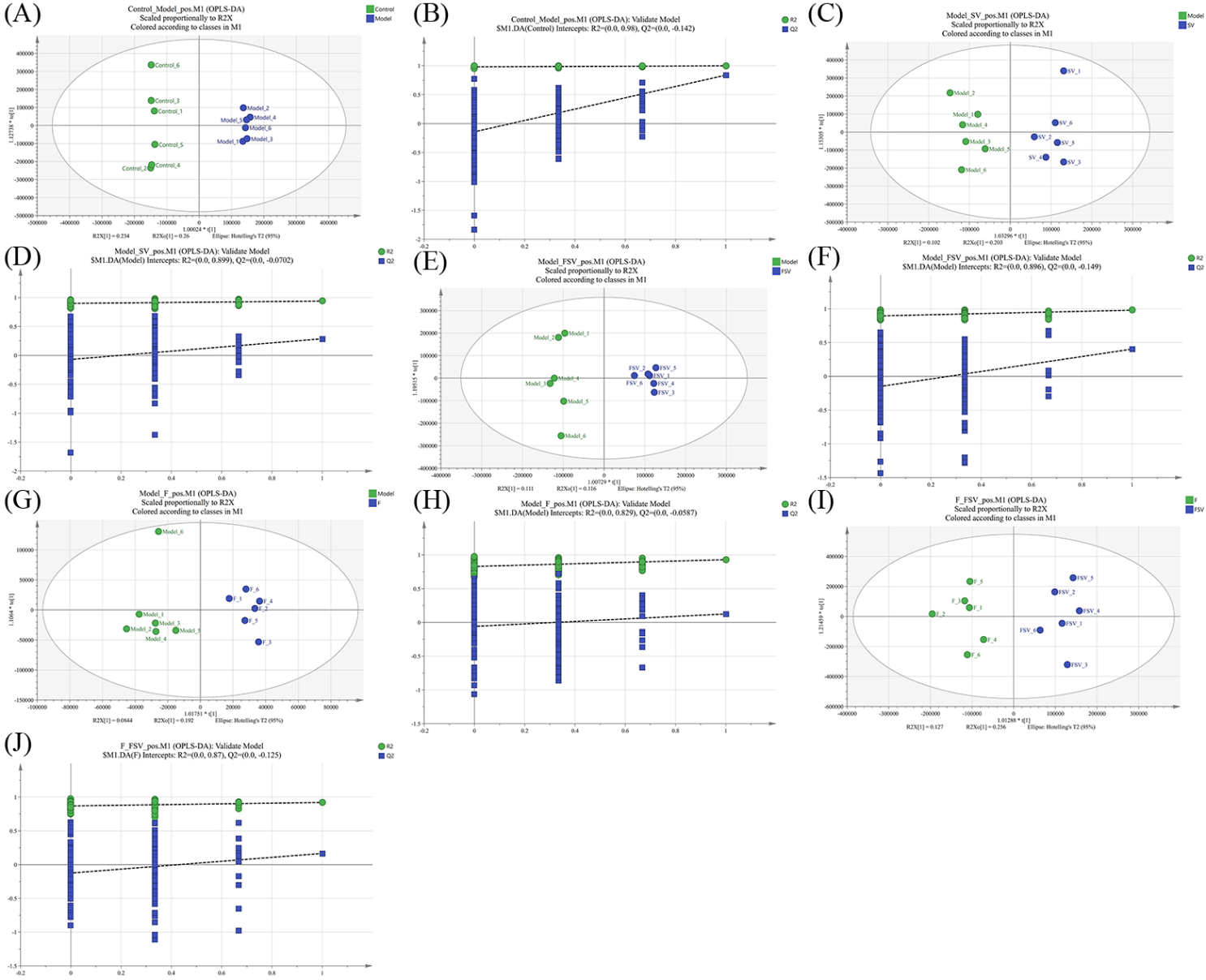


**Supplementary Figure 4.** Comprehensive orthogonal partial least squares-diacriminant analysis (OPLS-DA) in the positive ion mode. (A) Permutation test diagram between Control and Model group; (B) Score plot diagram between Control and Model group; (C) Permutation test diagram between SV and Model group; (D) Score plot diagram between SV and Model group; (E) Permutation test diagram between FSV and Model group; (F) Score plot diagram between FSV and Model group; (G) Permutation test diagram between F and Model group; (H) Score plot diagram between F and Model group; (I) Permutation test diagram between F and FSV group; (J) Score plot diagram between F and FSV group.


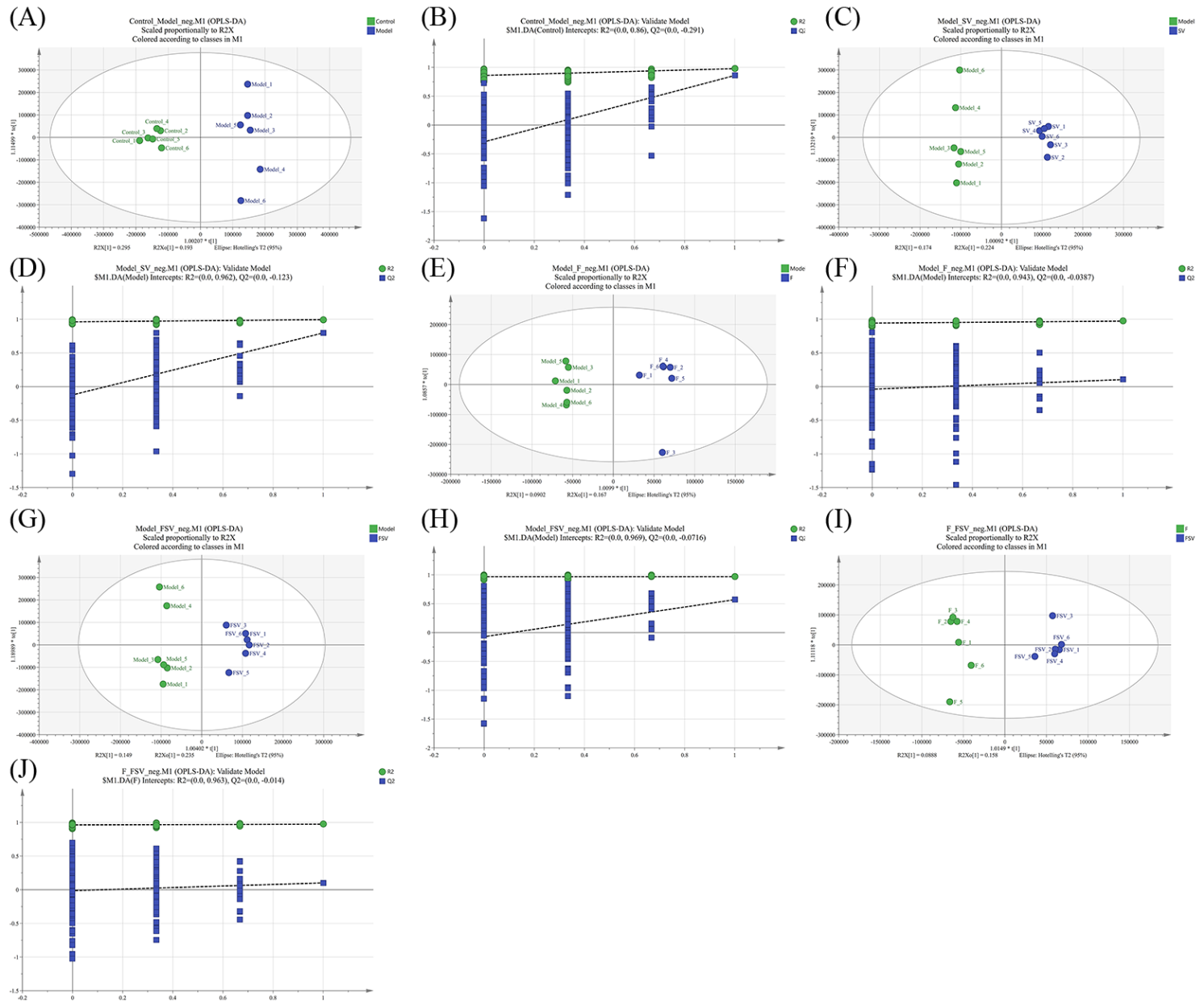


**Supplementary Figure 5.** Comprehensive OPLS-DA in the negative ion mode. (A) Permutation test diagram between Control and Model group; (B) Score plot diagram between Control and Model group; (C) Permutation test diagram between SV and Model group; (D) Score plot diagram between SV and Model group; (E) Permutation test diagram between F and Model group; (F) Score plot diagram between F and Model group; (G) Permutation test diagram between FSV and Model group; (H) Score plot diagram between FSV and Model group; (I) Permutation test diagram between F and FSV group; (J) Score plot diagram between F and FSV group.


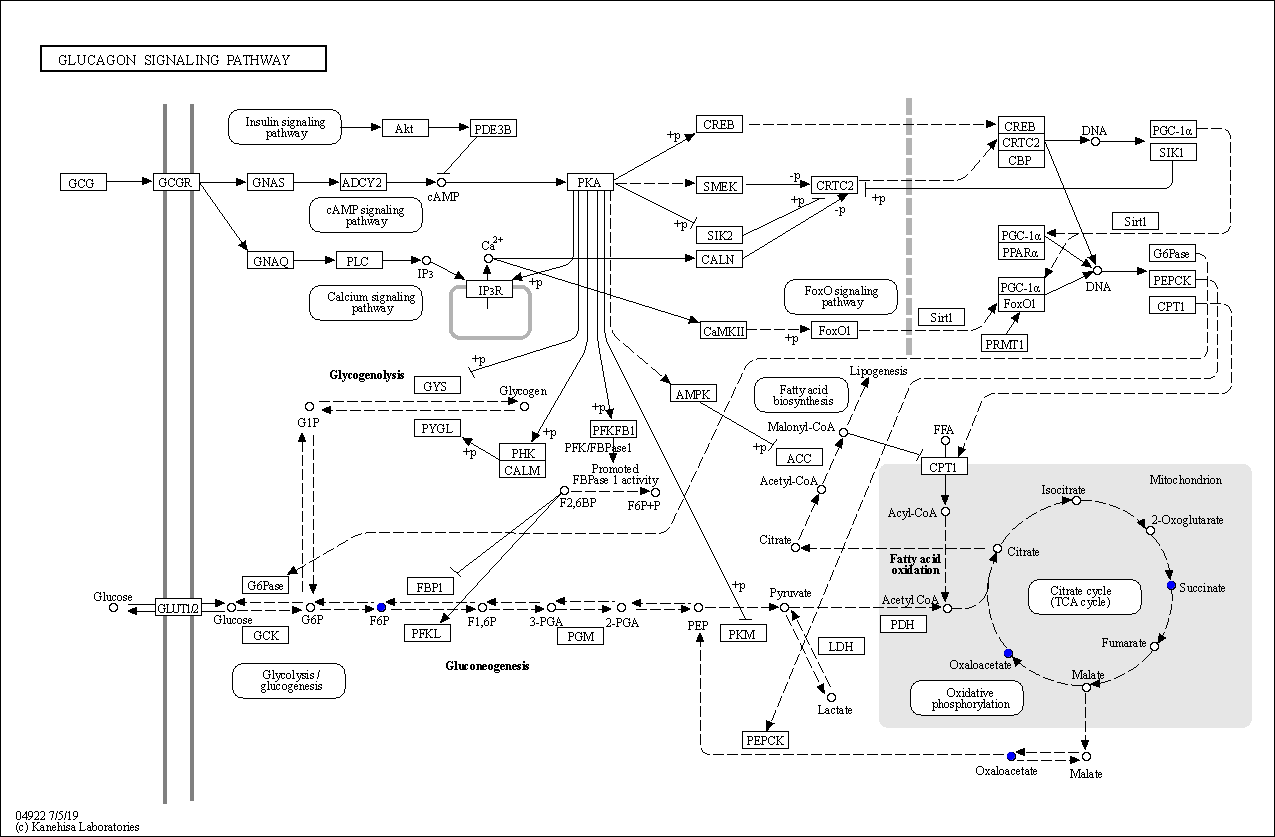


**Supplementary Figure 6.** Glucagon signaling pathway.

## Supplementary Tables

**Supplementary Table 1.** Detailed description of sample size estimation

| Endpoint | N | SS_between_ | SS_total_ | η^2^ | Cohen’s f |
| --- | --- | --- | --- | --- | --- |
| AUC | 6 | 0.9742 | 0.0258 | 37.7313 | 6.1426 |
| GSP | 6 | 0.9524 | 0.0476 | 20.0112 | 4.4734 |
| Serum T-CHO | 6 | 0.4941 | 0.5059 | 0.9768 | 0.9883 |
| Serum TG | 6 | 0.6182 | 0.3818 | 1.6190 | 1.2724 |
| Serum HDL-c | 6 | 0.5408 | 0.4592 | 1.1777 | 1.0852 |
| Serum LDL-C | 6 | 0.6621 | 0.3379 | 1.9593 | 1.3998 |
| F/B | 6 | 0.2745 | 0.7255 | 0.3784 | 0.6152 |

**Notes:** η^2^=SS_between_/SS_total_, Cohen/s f=Sqrt(η^2^). The Cohen's f values are all greater than 0.400, indicating that it is reasonable to analyze with 6 data points.

**Supplementary Table 2.** Comprehensive list of 22 active compounds of FSV.

| No | Name | ppm | Mass | Formula | Type | Class | KEGG |
| --- | --- | --- | --- | --- | --- | --- | --- |
| 1 | Tomentin | 0.835 | 346.07 | C_17_H_14_O_8_ | [M-H]- | Flavonoids | C04581 |
| 2 | Apigenin | 4.295 | 270.05 | C_15_H_10_O_5_ | [M-H]- | Flavonoids | C01477 |
| 3 | 3,7-Dimethylquercetin | 1.905 | 330.07 | C_17_H_14_O_7_ | [M-H]- | Flavonoids | C01265 |
| 4 | Honyucitrin | 4.890 | 406.18 | C_25_H_26_O_5_ | [M+H]+ | Flavonoids | / |
| 5 | Tangeritin | 3.823 | 372.12 | C_20_H_20_O_7_ | [M+H]+ | Flavonoids | C10190 |
| 6 | Demethylnobiletin | 0.484 | 388.12 | C_20_H_20_O_8_ | [M+H]+ | Flavonoids | / |
| 7 | Apigenin 7,4’-dimethyl ether | 4.666 | 298.08 | C_17_H_14_O_5_ | [M+H]+ | Flavonoids | C10019 |
| 8 | Acacetin | 2.848 | 284.07 | C_16_H_12_O_5_ | [M-H]- | Flavonoids | C01470 |
| 9 | Tetramethylscutellarein | 2.650 | 342.11 | C_19_H_18_O_6_ | [M+H]+ | Flavonoids | C14472 |
| 10 | Isorhamnetin | 0.393 | 316.06 | C_16_H_12_O_7_ | [M-H]- | Flavonoids | C10084 |
| 11 | Jaceidin | 1.689 | 360.084 | C_18_H_16_O_8_ | [M+H]+ | Flavonoids | / |
| 12 | 6-Methoxy-7-methylquercetin | 2.639 | 346.07 | C_17_H_14_O_8_ | [M+HCOO]- | Flavonoids | C17788 |
| 13 | FA 18_4;O | 0.647 | 292.20 | C_18_H_28_O_3_ | [M+H]+ | Fatty acids | C16320 |
| 14 | (2’E,4’Z,8E)-Colneleic acid | 0.742 | 294.22 | C_18_H_30_O_3_ | [M-H_2_O+H]+ | Fatty acids | C19827 |
| 15 | 4-Hydroxyretinoic acid | 1.553 | 316.20 | C_20_H_28_O_3_ | [M+K]+ | Retinoids | C16677 |
| 16 | Tetrahydropalmatine | 1.116 | 355.18 | C_21_H_25_NO_4_ | [M-NH_3_-H]- | Alkaloids | C02890 |
| 17 | (S)-scoulerine | 2.248 | 327.15 | C_19_H_21_NO_4_ | [M+NH_4_]+ | Alkaloids | C02106 |
| 18 | Autumnaline | 2.633 | 373.19 | C_21_H_27_NO_5_ | [M-NH_3_+H]+ | Alkaloids | C16707 |
| 19 | Promethazine | 1.240 | 284.13 | C_17_H_20_N_2_S | [M+H]+ | Promethazine | C07404 |
| 20 | Diethylstilbestrol | 1.957 | 268.15 | C_18_H_20_O_2_ | [M+H]+ | Diethylstilbestrol | C07620 |
| 21 | cis-Hinokiresinol | 1.781 | 252.12 | C_17_H_16_O_2_ | [M-H]- | Phenylpropanoids | C10628 |
| 22 | 16-oxo-palmitate | 0.347 | 270.22 | C_16_H_30_O_3_ | [M-H]- | Fatty acids | C19614 |

**Supplementary Table 3.** ADME information and content changes of intersecting metabolites

| No | Name | GI absorption | BBB permeant | P-gp substrate | Lipinski | Ghose | Veber | Egan | Muegge | Up or down |
| --- | --- | --- | --- | --- | --- | --- | --- | --- | --- | --- |
| 1 | 1,2-Dihydronaphthalene-1,2-diol | High | Yes | No | Yes | Yes | Yes | Yes | No | Down |
| 2 | 2-Pentanone | High | Yes | No | Yes | No | Yes | Yes | No | Down |
| 3 | 4-amino-4-deoxychorismate | High | No | No | Yes | Yes | Yes | Yes | No | Down |
| 4 | 4-Hydroxyphenylpyruvic acid | High | No | No | Yes | Yes | Yes | Yes | No | Up |
| 5 | 6-Methylsalicylic acid | High | Yes | No | Yes | No | Yes | Yes | No | Down |
| 6 | AICA-riboside | Low | No | No | Yes | No | No | No | No | Up |
| 7 | Androstenedione | High | Yes | No | Yes | Yes | Yes | Yes | Yes | Down |
| 8 | D-Ribose | Low | No | No | Yes | No | Yes | Yes | No | Up |
| 9 | L-Lysine | High | No | No | Yes | No | Yes | Yes | No | Down |
| 10 | Morphine-3-glucuronide | Low | No | Yes | Yes | No | No | No | No | Down |
| 11 | Pyroglutamic acid | High | No | No | Yes | No | Yes | Yes | No | Up |
| 12 | Salvigenin | High | Yes | No | Yes | Yes | Yes | Yes | Yes | Down |
| 13 | Spiramine A | High | Yes | Yes | Yes | Yes | Yes | Yes | Yes | Down |
| 14 | 1-Dehydroprogesterone (ST 21_4;O2) | High | Yes | No | Yes | Yes | Yes | Yes | Yes | Down |
| 15 | Succinic acid | High | No | No | Yes | No | Yes | Yes | No | Down |
| 16 | Tyramine | High | Yes | No | Yes | No | Yes | Yes | No | Down |

Notes: “Up” indicates that the metabolite content increased in the liver of the FSV group with T2DM, while “Down” indicates the opposite result.
